# Graph neural network modeling of pedestrian space vitality in campus waterfront space: a Flow–Dwell perspective for design translation

**DOI:** 10.3389/fpubh.2026.1963816

**Published:** 2026-10-29

**Authors:** Zhiye Yu, Ying Bao, Shijie Li, Xiao Liu, Yue Pei, Bao-Jie He

**Affiliations:** 1 State Key Laboratory of Subtropical Building and Urban Science, School of Architecture, South China University of Technology, Guangzhou, China; 2 Architectural Design & Research Institute, South China University of Technology, Guangzhou, China; 3 School of Electrical Engineering and Computer Science, KTH Royal Institute of Technology, Stockholm, Sweden; 4 Energy Saving Technology Research Institute, South China University of Technology, Guangzhou, China; 5 Centre for Climate–Resilient and Low–Carbon Cities, School of Architecture and Urban Planning, Key Laboratory of New Technology for Construction of Cities in Mountain Area, Ministry of Education, Chongqing University, Chongqing, China; 6 School of Architecture, Design and Planning, The University of Queensland, Brisbane, QLD, Australia

**Keywords:** built environment, graph neural network, pedestrian space vitality, public health, spatial heterogeneity, waterfront space, Wi-Fi probe

## Abstract

In campus landscapes, waterfront pedestrian spaces support both movement and staying activities and may contribute to activity- and health-supportive environments. This study examined the associations between built-environment features and waterfront pedestrian-space vitality across 42 spatial units at two campuses of the South China University of Technology (20 at Wushan Campus and 22 at Guangzhou International Campus). Wi-Fi probe data were collected during weekday and weekend observation periods on December 11–14, 2025 (Wushan Campus) and December 18–21, 2025 (Guangzhou International Campus) to derive two device-based vitality proxies: Flow for transient activity and Dwell for staying-oriented activity, distinguished using unit-specific traversal-time thresholds. A Flow–Dwell quadrant framework was used to classify the spatial contexts, and graph neural network node regression was employed to incorporate spatial adjacency relationships. Feature perturbation was further used to quantify the model-prediction sensitivity at the node level and to support design interpretation. The results showed that (1) Flow and Dwell exhibited distinct spatial distributions and environmental sensitivity patterns; (2) Flow was better predicted by graph neural networks than Dwell (best overall *R*^2^ = 0.48, versus 0.04 for Dwell), whereas Dwell showed a substantially weaker model fit; and (3) the selected GNNs outperformed the best-performing conventional baselines for both targets, reducing the RMSE by 36.2% for Flow and 14.0% for Dwell. This study provides a spatially explicit framework for interpreting campus waterfront vitality and translating model-derived sensitivity patterns into design priorities, offering evidence for planning activity-supportive campus environments, and contributing to public-health-oriented spatial design.

## 1 Introduction

Under changing higher education pedagogies, student learning has extended beyond traditional lecture-based settings to include informal and collaborative activities (1). Accordingly, campus landscapes are evolving from single-purpose leisure areas to hybrid environments that support learning, interaction, and collaboration, while accommodating diverse forms of everyday activity and social engagement. This broader role makes campus landscapes increasingly relevant to public-health-oriented planning and the creation of activity-supportive environments. Previous studies have associated the architectural, landscape, and facility conditions of campus common spaces with students’ psychological restoration and related patterns of space use (2, 3).

Within campus environments, water bodies represent key landscape and ecological elements, regulating microclimate, enhancing environmental quality (4), and strengthening sense of place. Microclimatic conditions, in turn, influence outdoor-space use because heat exposure may constrain outdoor activities (5). Owing to their environmental comfort and visual appeal, waterfront areas attract a range of staying, recreation, and social interaction activities (6–8). Research on university common spaces has also associated perceived restorativeness with green-view exposure, waterscape coverage, and openness of spatial enclosures (9). In addition to accommodating these activities, pedestrian spaces formed around water bodies serve basic circulation functions and connect different functional areas through continuous landscape interfaces and spatial organization (10). Therefore, waterfront pedestrian spaces within campus landscape systems exhibit coupled “path” and “place” attributes, simultaneously functioning as essential components of daily movement networks and key interfaces for campus public life and social interaction (11), making their vitality relevant not only to spatial performance but also to the capacity of campus environments to support everyday activity and social use.

With ongoing campus development, the environmental quality of some waterfront pedestrian spaces has been negatively affected by vehicular interference, particularly traffic-related noise disturbances, which significantly influence pedestrians’ environmental perception and experience, leading to a decline in spatial experience and usage efficiency (12, 13). However, campus waterfront landscape spaces are largely developed based on conventional functional paradigms, which hinders their ability to accommodate diverse and overlapping activity demands. An increasing mismatch between spatial provision and behavioral needs highlights the need for targeted optimization (14).

However, current practice still relies on relatively uniform and coarse-grained interventions, resulting in a fundamental mismatch between complex objectives and simplified responses. Specifically, pedestrian-oriented public-space regeneration requires a balance between movement efficiency, environmental quality, and diverse activities. Moreover, pedestrian-oriented public spaces often exhibit clear spatial heterogeneity across segments, with variations in usage patterns and activity characteristics (15–17). Such spatial heterogeneity is not adequately addressed by current strategies, limiting their adaptability and capacity to support diverse movement, stay, and social-use needs that are relevant to public-health-oriented spatial planning.

The first challenge in campus-space renewal concerns the behavioral differentiation of pedestrian-space vitality. Recent studies have begun to distinguish between different activity patterns rather than treating vitality as a behaviorally homogeneous construct (18, 19). Movement-based activity patterns involve through-movement along paths and emphasize spatial continuity and movement efficiency. In contrast, staying-based activities include dwelling, leisure, and interaction and depend more strongly on spatial quality and environmental perception (20). These activity types differ in behavioral intent, duration, and spatial requirements. This distinction is also relevant from a public-health perspective, as campus-restoration studies have separately examined dynamic activity, static leisure, contact with nature, and social interaction as forms of space use associated with psychological restoration (2, 3).

Second, the effects of built-environment factors on pedestrian-space vitality are highly context-dependent. Existing studies typically describe the spatial environment in terms of circulation conditions, environmental perception, landscape resources, and activity support (21, 22). However, inconsistency in study findings (23–25), which can arise from differences in research objects and methods, also reflects the context-sensitive nature of environmental influences, which can vary substantially in both direction and magnitude depending on spatial features or dominant activity types (15, 26–28). Therefore, identifying such context dependence is important for avoiding uniform design prescriptions and matching environmental interventions to different patterns of spatial use.

Third, pedestrian-oriented spaces typically comprise morphologically and functionally interconnected paths and nodes that are spatiotemporally integrated and continuous (15, 29). Pedestrian perception and behavioral decision-making are not determined solely by the local features of individual spatial units but instead emerge dynamically through continuous spatial experience. Consequently, adjacent units often exhibit interdependencies in usage intensity, activity type, and spatiotemporal distribution (15). In this context, treating spatial units independently may fail to capture actual use patterns.

Common methods for determining the relationship between pedestrian-space vitality and the built environment include correlation analyses and regression models, which identify linear statistical associations between variables (30, 31). With advances in data availability and computational capacity, machine learning approaches are increasingly employed to better capture high-dimensional features and complex nonlinear relationships (19). Graph-based methods extend this capacity by representing spatial units as nodes and explicitly encoding their structural relationships (32). To address this spatial dependence, we employed graph neural networks (GNNs) for node-level regression (33). Spatial units are represented as nodes, whereas graph edges encode adjacency relationships that allow neighboring spatial conditions to be directly incorporated into the vitality prediction.

However, existing research on the relationship between pedestrian-space vitality and the built environment has predominantly focused on urban streets or commercial spaces (19, 34, 35), with less attention to campus pedestrian spaces that simultaneously accommodate movement and staying activities (36). In addition, most studies operationalize vitality using aggregate indicators (37–39), which may obscure differences in the environmental associations of distinct spatial-use patterns. Methodologically, conventional regression models provide useful estimates of global associations, and related spatial-regression models can account for specific forms of spatial dependence (36). Geographically weighted regression (GWR) can further characterize location-specific coefficient variations but relies on repeated local regressions, and recent studies have shown that local coefficient estimates can become unstable when the number of observation locations or effective local sample size is small (40, 41). Given that each campus in this study contained only 20–22 spatial units, GWR was not included as a benchmark.

This study does not assume that GNNs are inherently superior to conventional approaches. Instead, mean prediction, ordinary least-squares regression, and spatial-lag models were used as conventional baselines to compare the predictive performance under the same predefined data splits. In addition, although machine learning approaches offer predictive flexibility, their “black-box” nature may limit interpretation and design translation (42–44). To improve interpretability, this study combined a GNN with feature perturbation analysis to identify context-specific feature sensitivities and support spatial design interpretation.

This study examined the associations between built-environment features and pedestrian-space vitality in campus waterfront environments. By distinguishing between movement- and staying-oriented spatial-use patterns and relating them to environmental conditions, this study focuses on how campus spaces may better support everyday activities and social use. The Wi-Fi-derived indicators capture device-based patterns of space use and persistence that may inform the planning of accessible and activity-supportive campus environments; they should therefore be interpreted as spatial-use proxies rather than direct measures of physical activity, mental health, restorative experience, or other health outcomes.

Specifically, this study aimed to determine (1) how Wi-Fi-derived Flow and Dwell proxy indicators vary across campus waterfront spatial units; (2) which built-environment features show the greatest model-prediction sensitivity for Flow and Dwell, and how their relative sensitivity rankings vary across Flow–Dwell quadrants; and (3) how context-specific feature sensitivities identified through adjacency-aware GNN modeling and feature perturbations can inform multilevel spatial optimization priorities. These aims link spatial-use differentiation, environmental associations, and design translations within a continuous spatial framework.

The contributions of this study are as follows. First, it distinguishes Flow and Dwell as separate Wi-Fi-derived proxies of transient and staying-oriented spatial use, rather than treating vitality as a single aggregate construct. Second, it applies an adjacency-aware GNN framework to model nonlinear node-level associations while explicitly representing the relationships among neighboring spatial units. Third, it develops a multilevel design translation framework that uses Flow–Dwell quadrants to identify whether spatial units should prioritize Flow or Dwell enhancement and then combines node-level sensitivity rankings with existing environmental conditions to determine targeted built-environment optimization priorities. Its contribution to public health lies at the environmental-support level by informing the design of campus spaces that better support everyday activities and social use, rather than by estimating health outcomes directly.

## 2 Methods

An analytical framework was developed to examine the relationships between built-environment features and the vitality of campus waterfront pedestrian spaces. This framework comprised four components: spatial unit delineation, dataset construction, modeling, and design translation (Figure 1). In the modeling stage, Flow and Dwell indicators are analyzed within a behavioral quadrant framework, and a GNN is employed to examine context-dependent associations across spatial conditions. Finally, the analytical findings are integrated with spatial contexts to derive refined optimization strategies.

**Figure 1:**
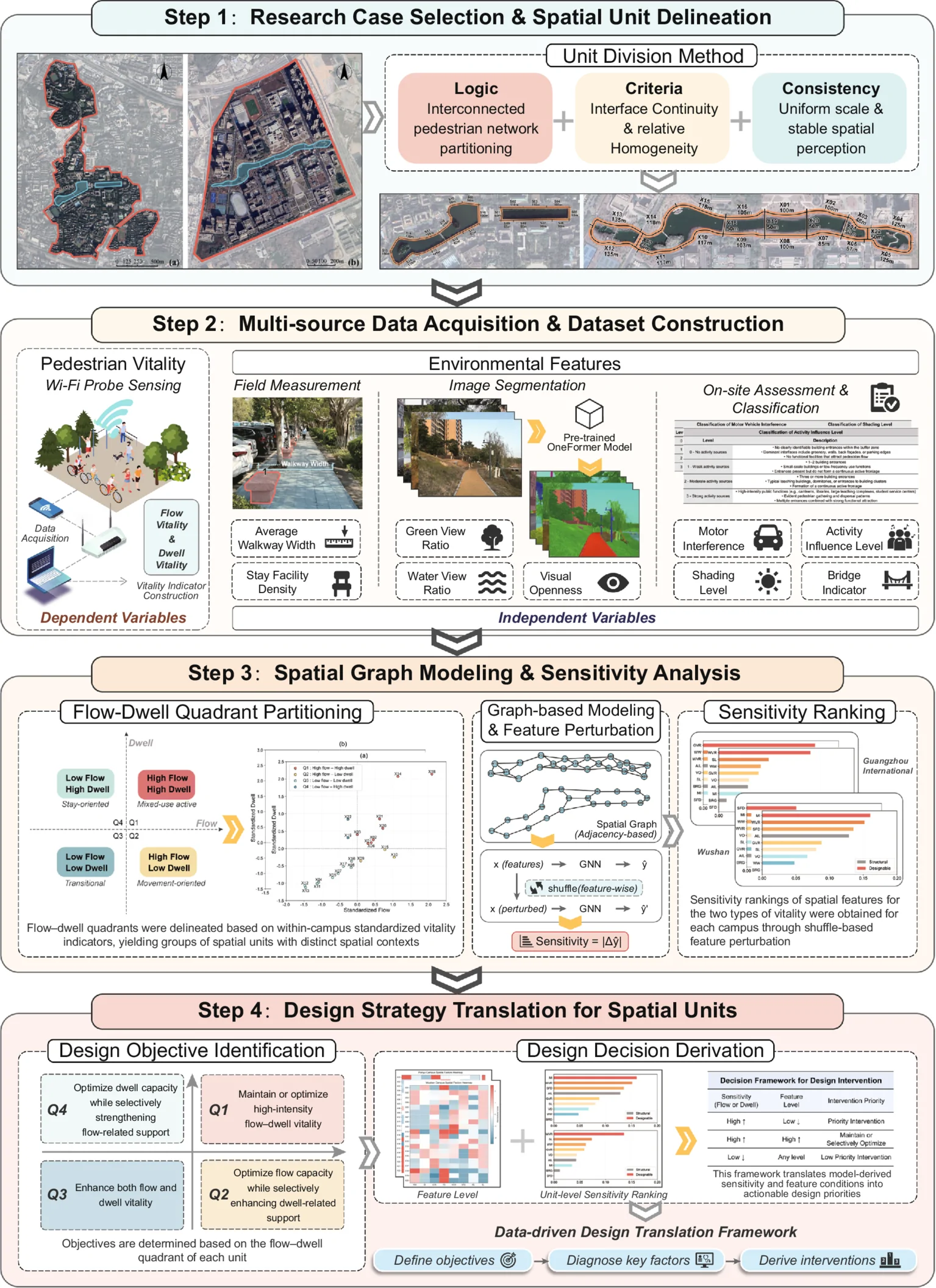
Schematic of the proposed analytical framework, comprising spatial delineation, dataset construction, modeling, and design translation.

### 2.1 Study area and spatial unit delineation

This study involved the pedestrian spaces surrounding core water bodies at the Wushan Campus (WSC) and Guangzhou International Campus (GZIC) of the South China University of Technology, Guangdong, China (Figure 2). These spaces exhibit both flow and dwell characteristics and are continuously distributed along the waterfront, representing some of the most active and behaviorally diverse areas on the campus. WSC and GZIC represent mature and newly developed campuses, respectively, which differ significantly in spatial morphology, water system structure, and pedestrian system configurations, while sharing comparable educational functions and campus-use contexts (Table 1). This combination of “structural differences and functional consistency” provides a useful basis for comparative analyses.

**Figure 2:**
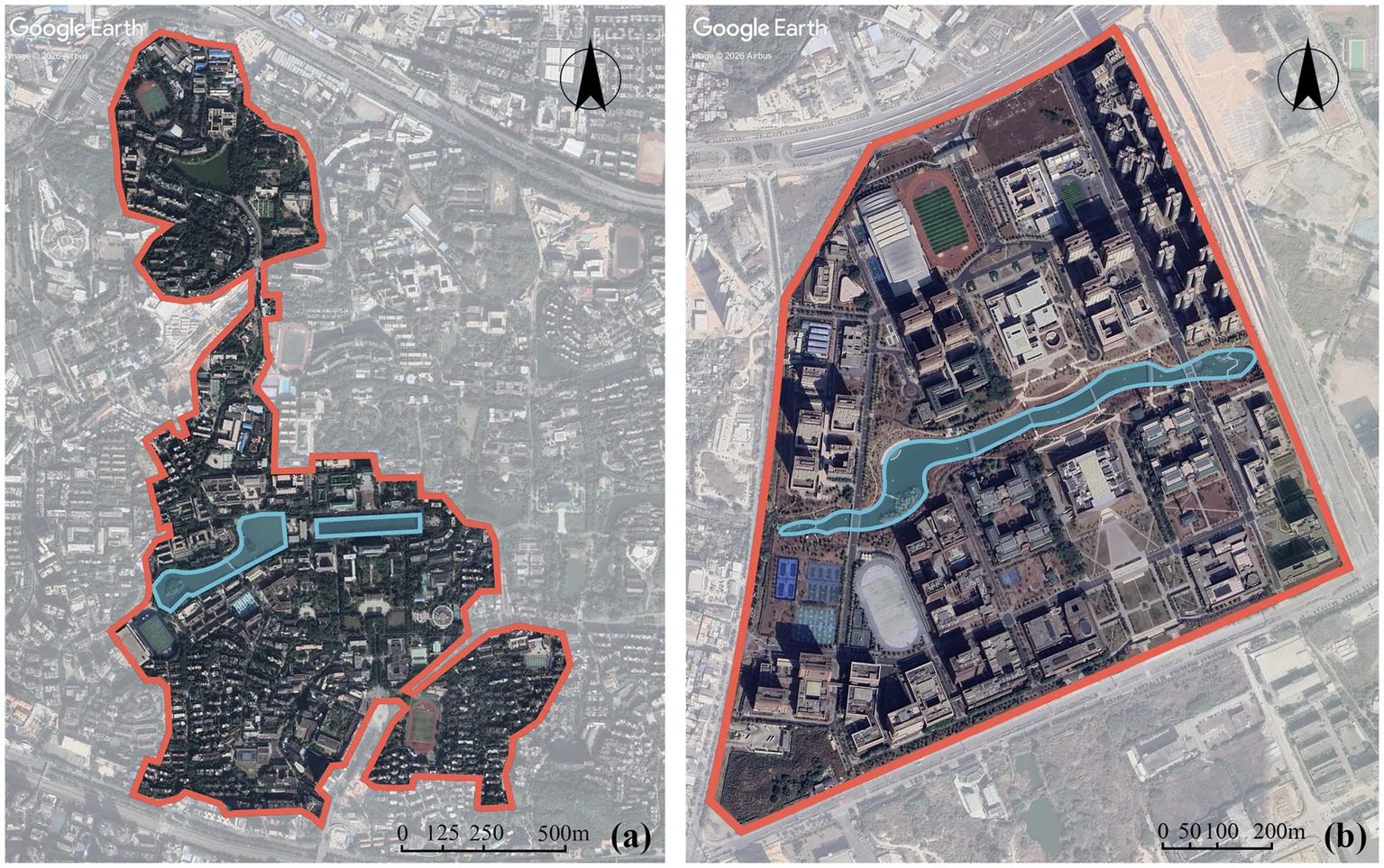
Maps showing the study area outlines and presence of water bodies: **(a)** Wushan Campus (WSC) and **(b)** Guangzhou International Campus (GZIC).

**Table 1:** Comparison of basic characteristics between WSC and GZIC.

Campus	Year of establishment	Development stage	Morphological type	Spatial density	Functional integration	Water system form	Pedestrian interface
Wushan Campus	1934	Mature	Axial-cluster hybrid structure	High	Mixed academic-residential functions	Discontinuous linear water system	Direct waterfront pedestrian system
Guangzhou International Campus	2022	Newly developed	Cluster-based structure	Medium	Spatially segregated academic and residential functions	Continuous linear water system	Buffered waterfront pedestrian system

On both campuses, the water bodies were located within the core spatial structure and closely aligned with the primary spatial axes, resulting in high levels of pedestrian aggregation. Variations in spatial organization patterns and built-environment configurations between WSC and GZIC support a context-dependent analysis of the associations between environmental features and pedestrian-space vitality.

To support node-level comparisons and graph-based modeling, the continuous waterfront pedestrian spaces were further divided into interconnected spatial units.

Network- or unit-based partitioning approaches can effectively capture the relationships between spatial structures and pedestrian activity (45). To ensure the stability of built-environment features at the unit level, spatial units were delineated according to the principles of interface continuity and relative internal homogeneity. Specifically, while maintaining comparable unit sizes, each unit was defined to preserve internal consistency in environmental features and perceptual conditions (12, 46). A WSC unit that was under maintenance during the observation period was not included in the 20 WSC units retained for modeling (Figure 3).

**Figure 3:**
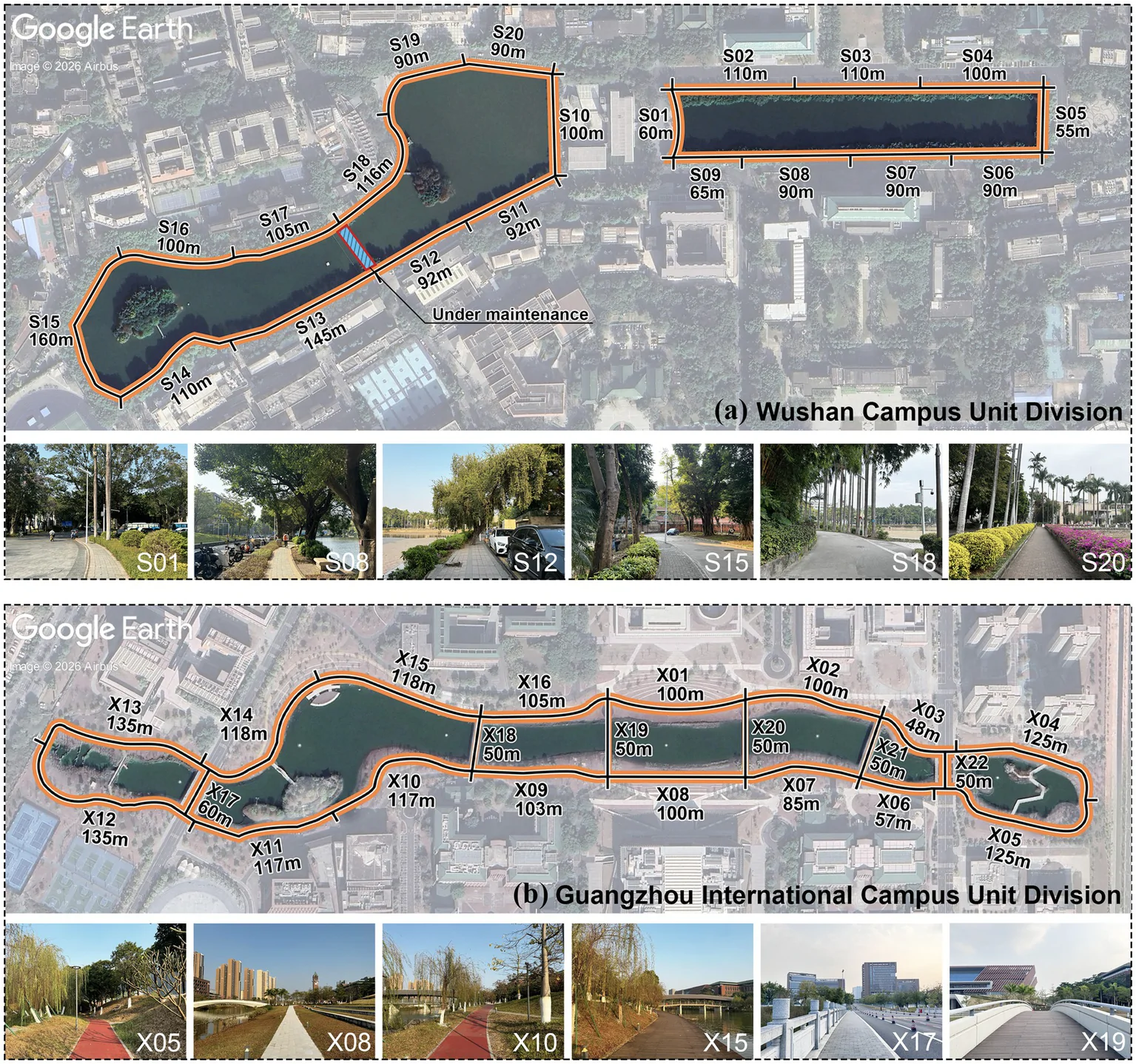
Spatial-unit delineation and representative photographs for **(a)** WSC and **(b)** GZIC.

### 2.2 Dataset construction

#### 2.2.1 Dependent variables: flow and dwell indicators of pedestrian-space vitality

Wi-Fi probe sensing has increasingly been used to characterize pedestrian presence, flow, and space-use patterns in public environments because it enables continuous and non-intrusive detection of Wi-Fi-enabled mobile devices (47–49). Previous studies have emphasized that such measurements are more appropriate for identifying relative patterns and spatiotemporal variations than for representing exact pedestrian counts. The probes recorded anonymized device IDs, timestamps, and received signal strength indicator (RSSI) values. Data were collected during two afternoon periods: 14:30–15:30 and 16:00–17:00. Campus outdoor activity and pedestrian occupancy vary substantially with the time of day and academic schedules, including class-transition periods (50, 51). Accordingly, the observation periods were selected with reference to the campus timetable and field reconnaissance to avoid pronounced tidal flows associated with class changes and to capture the relatively discretionary patterns of walking, recreation, and staying. The morning periods were excluded based on field reconnaissance, indicating fewer consistent opportunities to observe discretionary activities outside of class-transition peaks.

Fieldwork was conducted from December 11 to 14, 2025, at WSC and from December 18 to 21, 2025, at GZIC. Each spatial unit was observed during the selected periods on a weekday and weekend. All surveys were conducted under comparable dry weather conditions, with sunny to partly cloudy skies and temperatures of approximately 18 °C. The sampling interval was set to 5 s. This design was intended to compare the relative spatial differences under comparable observation conditions rather than to represent full-day, weekly, seasonal, or long-term vitality patterns.

Six TZ-4007 Wi-Fi probes, with a nominal signal coverage range of approximately 50–100 m, were used. During each survey round, probes were installed approximately 1.2 m above the ground level. To reduce the potential overlap between simultaneously operating probes, approximately three to four spatial units were deployed apart from the adjacent units. Because effective Wi-Fi coverage can vary with local obstructions and environmental conditions, the complete uniformity of signal coverage cannot be guaranteed.

Raw data pre-processing included the following steps: (1) removal of invalid records and abnormal entries; (2) filtering by signal strength, excluding records with RSSI values ≤−85 dBm, following empirical thresholds used in previous Wi-Fi localization and fingerprinting studies, thereby reducing detections more likely to originate outside the target spatial unit and limiting weak-signal interference between nearby measurement areas (52, 53); and (3) organization of detections by spatial unit, 20-min time window, and device ID, while retaining repeated timestamped detections for calculating device-level observed duration.

The detected device identifiers were not globally deduplicated across spatial units or observation sessions because detections recorded in different units or sessions were treated as distinct spatial-use observations. Only duplicate raw records were removed during pre-processing. Separate probe deployment and RSSI filtering were used to reduce simultaneous cross-unit attribution, although residual overlap could not be completely excluded. Moreover, Wi-Fi probes detect mobile devices rather than individuals, and devices with Wi-Fi that are disabled or otherwise undetectable are not captured. Accordingly, the resulting indicators were interpreted as relative device-based patterns of spatial use, rather than exact pedestrian counts or direct observations of individual behavior.

Previous studies have characterized spatial vitality using the complementary dimensions of pedestrian movement intensity and staying duration or persistence (19, 54, 55). Accordingly, two device-based indicators were constructed in this study: Flow, which represents transient device activity, and Dwell, which represents the staying-oriented device activity. Rather than representing the full multidimensional construct of pedestrian-space vitality, these indicators capture two behaviorally distinct aspects of detected spatial use. For formal representation, i denotes the spatial unit and w denotes the time window. Each 1-h observation period was divided into three equal 20-min windows, within which two indicators were calculated. $M_{i,w}$ denotes the set of unique devices detected in the spatial unit i during the time window w and $m\in M_{i,w}$ represents an individual device.

(1) Flow

Flow represents the transient device activity within a spatial unit. For each detected device m , the observed duration ${T_{i,w}}^{\left(m\right)}$ was calculated as the interval between the first and last detections within the spatial unit i and time window w . To account for differences in unit length, a unit-specific behavioral threshold was defined based on the theoretical traversal time (56), where $L_{i}$ denotes the length of spatial unit i and the reference walking speed was set to v=1.4m/s (31, 57). The expected traversal time is calculated as follows:

$$T_{i}=\frac{L_{i}}{v}$$

Considering the variability in the actual walking speed, a tolerance factor of 1.5 was applied, yielding a transient threshold of 1.5 $T_{i}$ . Therefore, flow is defined as the number of devices exhibiting transient activity within the spatial unit i during the time window w (Equation 1):

$$\mathrm{Flo}w_{i,w}=\sum_{m\in M_{i,w}}1(T_{i,w}^{\left(m\right)}\leq 1.5T_{i})$$ (1)

(2) Dwell

Dwell represents staying-oriented device activity within a spatial unit. Devices whose observed duration exceeded the unit-specific traversal-time threshold were classified as Dwell, indicating device persistence beyond the expected traversal time rather than transient passage. Dwell was calculated using Equation 2:

$$\text{Dwel}l_{i,w}=\sum_{m\in M_{i,w}}1(T_{i,w}^{\left(m\right)}>1.5T_{i})$$ (2)

By construction, Flow and Dwell were mutually exclusive within each spatial unit and time window and jointly partitioned all detected devices such that

$$\mathrm{Flo}w_{i,w}+\text{Dwel}l_{i,w}=|M_{i,w}|.$$

Flow and Dwell were averaged across three 20-min windows within each observation session. The resulting values were then standardized separately within each campus and day type using *z*-scores, after which the weekday and weekend standardized values were equally averaged to obtain the final unit-level indicators.

#### 2.2.2 Independent variables: built-environment features

Drawing on prior studies on the relationships between the built environment and public space behavior (16, 21, 23, 58, 59), and considering the specific characteristics of campus waterfront pedestrian spaces, four dimensions of built-environment features were selected: circulation conditions, environmental perception, visual landscape, and activity support (Table 2). Pedestrian behavior depends on the physical conditions supporting movement, but is also influenced by microscale environmental comfort and potential disturbances, which together shape the extent to which campus spaces can support everyday walking and staying activities and contribute to a health-supportive environment (4, 38). In addition, streetscape visual features (e.g., green view ratio and visual openness) are significantly associated with spatial vitality (46), partly by shaping visual perception and experiential quality (60). Finally, surrounding functions and activity sources can contribute to pedestrian activity and spatial vitality (61). Accordingly, nine built-environment features were used to characterize the spatial environment. Three data acquisition approaches were employed: field measurement, image segmentation, and on-site assessment.

(1) Construction of ordinal variables

**Table 2:** Data acquisition methods, data types, and abbreviations for built-environment features used in this study.

Data acquisition method	Variable type	Built-environment feature	Abbreviation	Description
Field measurement	Continuous	Average walkway width	WW	Average effective width available for pedestrian movement
		Stay facility density	SFD	Density of facilities supporting staying activities within the unit
Image segmentation	Continuous	Green view ratio	GVR	Proportion of vegetation within the visual field
		Visual openness	VO	Proportion of visible open space within the visual field
		Water view ratio	WVR	Proportion of water bodies within the visual field
On-site assessment	Ordinal	Motor interference	MI	Level of motor vehicle interference within the pedestrian space
		Shading level	SL	Degree of shading within the pedestrian space
		Activity influence level	AIL	Intensity of surrounding activity sources
	Binary	Bridge	BRG	Describes whether the unit is a bridge (0 = no, 1 = yes)

The motor interference, shading level, and activity influence level were classified into ordinal categories based on field surveys and spatial characteristics (Tables 3–5).

**Table 3:** Classification of motor interference level.

Level	Description
0	No motor vehicle interference (located away from traffic)
1	Adjacent to roadways but separated by vegetation buffers
2	Directly adjacent to traffic lanes or parking spaces
3	Significant pedestrian–vehicle interaction (shared space)

**Table 4:** Classification of shading level.

Level	Description
0	No shading
1	Intermittent shading provided by vegetation
2	Continuous shading from vegetation or light structures
3	Strong shading provided by permanent structures

**Table 5:** Classification of activity influence level.

Level	Description
0	No activity sources: no visible building entrances within the buffer zone; interfaces mainly consist of green spaces, walls, back façades, or parking edges; no facilities attracting pedestrian activity
1	Weak activity sources: 1–2 building entrances; small-scale buildings or low-frequency functions; entrances present but with no continuous active frontage
2	Moderate activity sources: ≥3 entrances; typical teaching buildings, dormitory entrances, or academic building clusters; continuous active frontage
3	Strong activity sources: high-intensity public functions (e.g., canteens, libraries, major teaching complexes, service centers); clear pedestrian congregation patterns; multiple entrances with strong functional attraction

For the activity influence level, the centerline of the waterfront pedestrian path was used as the reference baseline to account for variations in building setback distances across different campuses. Activity influence was assessed within two buffer ranges (30 and 60 m); the highest level identified across each range was assigned as the activity influence level for each spatial unit.

(2) Image-based variables

Semantic segmentation was performed on field photographs within each spatial unit using the OneFormer architecture (62). Based on segmented images, the proportions of vegetation, visible water, and open space within the pedestrian field of view [corresponding to the green view ratio (GVR), water view ratio (WVR), and visual openness (VO), respectively] were calculated as average pixel ratios (Figure 4). Field photographs were collected using a standardized protocol: images were captured along the full length of each spatial unit with approximately 15 evenly distributed sampling points along each spatial unit; all photographs were captured at a height of 1.5 m with a focal length of 28 mm to approximate pedestrian visual perception during normal walking; and images were collected under similar lighting conditions to ensure consistency.

**Figure 4:**
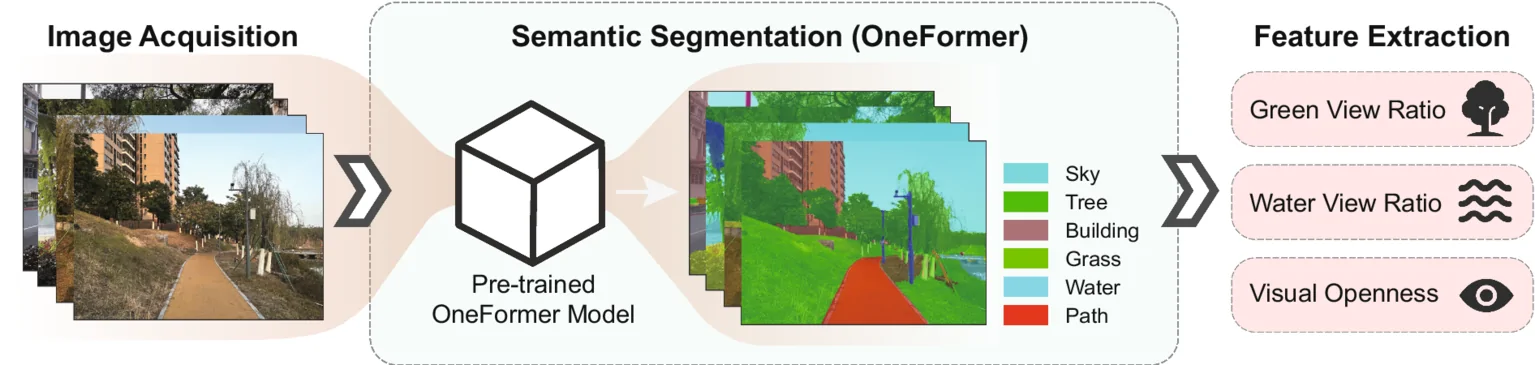
Semantic segmentation of field photographs based on OneFormer.

### 2.3 Data analysis methods

Following dataset construction, the analytical workflow comprised four main stages: (1) behavioral context classification of all spatial units using the Flow–Dwell quadrant framework, (2) graph construction and GNN-based node-level regression with architecture and hyperparameter selection, (3) benchmark comparison and robustness evaluation using conventional models and statistical reporting, and (4) feature perturbation and multilevel sensitivity analysis (Figure 5).

**Figure 5:**
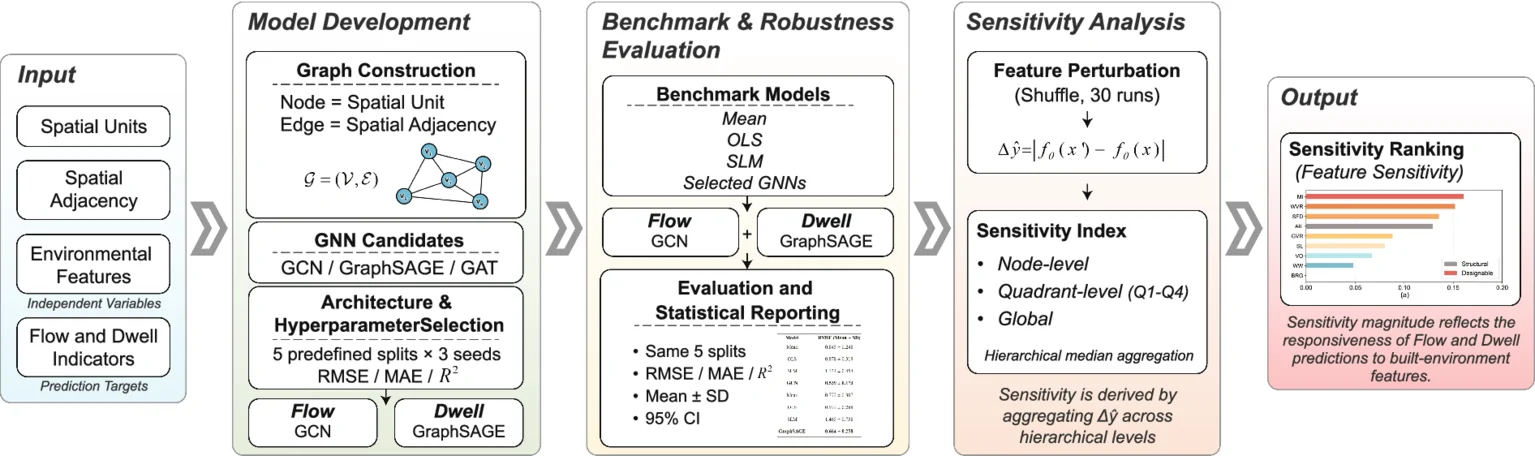
Schematic of the modeling, benchmark evaluation, and sensitivity-analysis workflow.

#### 2.3.1 Flow–Dwell quadrant classification

To further characterize spatial-use patterns across the spatial units of campus waterfront pedestrian spaces, a Flow-Dwell quadrant diagram was constructed based on standardized flow and dwell indicators (Figure 6). In this framework, the horizontal and vertical axes represent Flow and Dwell indicator values, respectively, yielding four spatial-use contexts: high flow–high dwell, high flow–low dwell, low flow–low dwell, and low flow–high dwell. Similar approaches have been adopted in previous studies, where combinations of multiple indicators were used to classify spatial-use patterns and reveal differences in spatial use (63, 64). This classification was not designed to evaluate the performance of spatial units but rather to identify their relative usage characteristics under existing spatial and environmental conditions. The Flow-Dwell quadrant framework serves as a contextual interpretive tool, linking identified usage patterns to context-specific optimization objectives and intervention priorities to inform targeted, unit-level spatial design strategies.

**Figure 6:**
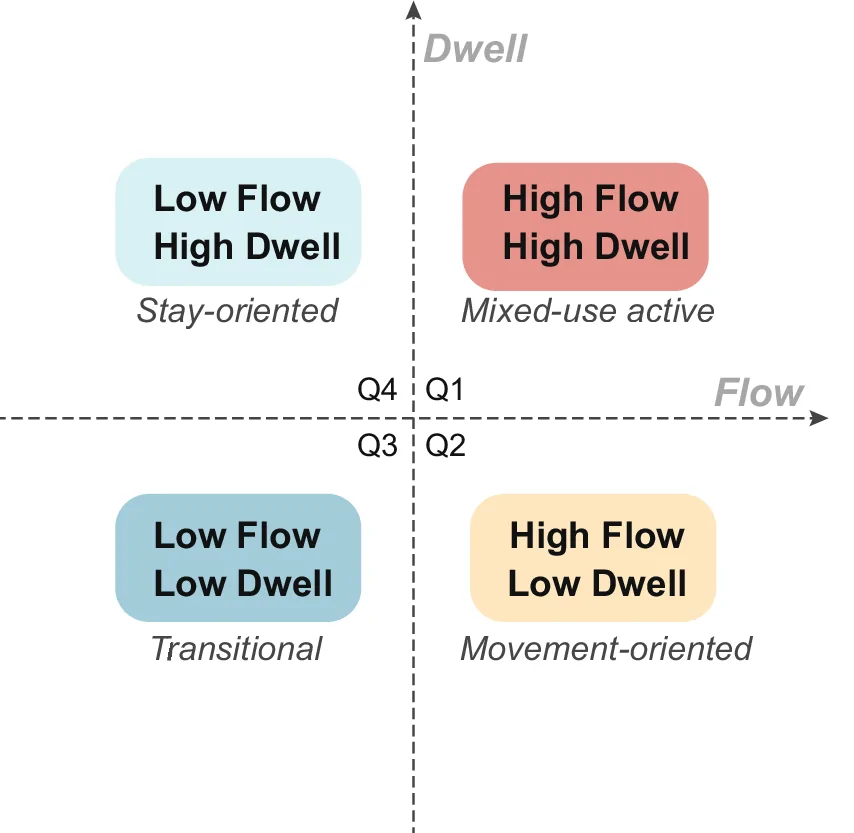
Flow–Dwell quadrant diagram.

#### 2.3.2 Graph construction and node-level regression

A graph structure was constructed based on the spatial adjacency relationships between delineated spatial units. The graph was defined as G=(V,E) , where $V=\{v_{1},v_{2},\cdots ,v_{n}\}$ denotes the set of nodes corresponding to spatial units and $E=\{(v_{1},v_{2}),(v_{1},v_{3}),(v_{1},v_{4}),\ldots \}$ denotes the set of edges representing the physical adjacency between neighboring units (Figure 7). An undirected edge is established between two nodes if the corresponding spatial units are directly adjacent, allowing continuous pedestrian movement. For compatibility with GNN implementations, each undirected edge was represented by a pair of directed edges.

**Figure 7:**
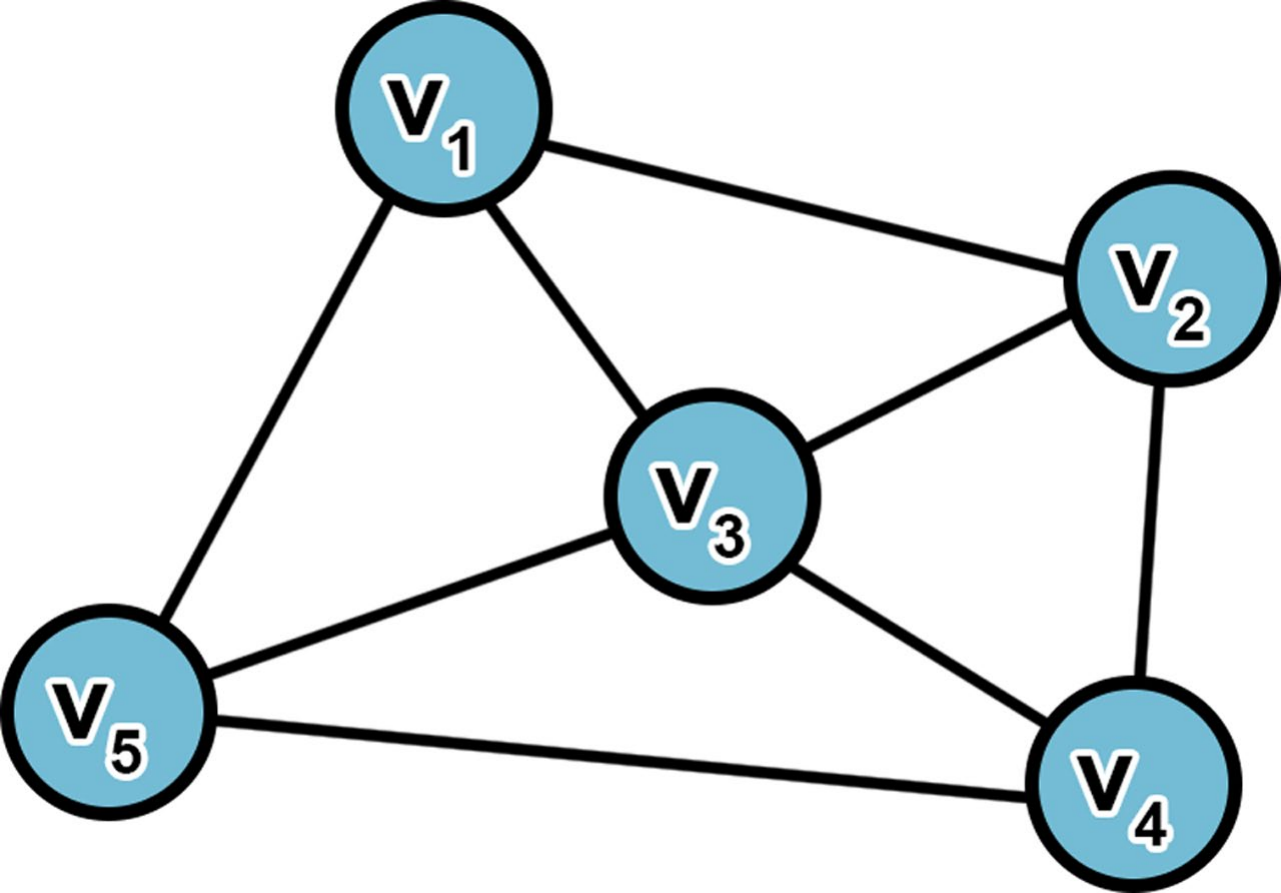
Schematic of the graph structure.

Built-environment features were used as the node attributes. The symbols for each spatial feature are presented in Table 6. For each node $v_{i}$ , the built-environment features were represented as the feature vectors $x_{i}\in {\mathbb{R}}^{9}$ and $x_{i}=(x_{i1},x_{i2},\ldots ,x_{i9})$ . Flow and Dwell were modeled separately as node-level regression targets, where $y_{i}\in \mathbb{R}\;$ denotes the observed Flow or Dwell value of node $v_{i}$ ; and $\hat{y_{i}}$ denotes the corresponding prediction. The regression process is expressed by Equation 3:

$$\hat{y_{i}}=f_{\theta }(x_{i},\{x_{j}|v_{j}\in N(v_{i})\})$$ (3)

where $f_{\theta }(\cdot )$ denotes the regression function learned by the GNN based on the graph structure and node features. Separate GNN models were trained for Flow and Dwell.

**Table 6:** Symbols of spatial elements (built-environment features).

$x_{\mathrm{ik}}$	$x_{i1}$	$x_{i2}$	$x_{i3}$	$x_{i4}$	$x_{i5}$	$x_{i6}$	$x_{i7}$	$x_{i8}$	$x_{i9}$
Feature	WW	SFD	GVR	VO	WVR	MI	SL	AIL	BRG

#### 2.3.3 GNN model selection and hyperparameter tuning

Considering the sensitivity of GNN performance to model architecture and hyperparameter settings under small-sample conditions, and drawing on previous studies of graph-based learning and spatiotemporal forecasting (65–67), we evaluated three representative architectures for Flow and Dwell node-level regression: Graph Convolutional Network (GCN), GraphSAGE, and Graph Attention Network (GAT).

Prior to model training, the input features were transformed and standardized according to their data types (Table 7). Continuous WW and SFD variables were log-transformed using Log1p before *z*-score standardization, whereas the remaining continuous and ordinal variables were standardized using *z*-scores, and the binary BRG variable was retained without transformation. All transformation and standardization parameters were estimated from the training data within each split and then applied to the corresponding validation data.

**Table 7:** Standardization methods for built-environment elements.

Built-environment feature	Feature type	Preprocessing
WW	Continuous	Log1p → *z*-score
SFD	Continuous	Log1p → *z*-score
GVR	Continuous	*z*-score
VO	Continuous	*z*-score
WVR	Continuous	*z*-score
MI	Ordinal	*z*-score
SL	Ordinal	*z*-score
AIL	Ordinal	*z*-score
BRG	Binary	No transformation

A reproducible random-search procedure was used for hyperparameter tuning. The common search space included hidden dimensions of 8, 16, 32, and 64; one to three GNN layers; dropout rates of 0.2, 0.3, and 0.5; learning rates of 0.001 and 0.005; and weight decay values of 0 and 5 × 10^−4^. For the GAT, the number of attention heads was selected as 1, 2, and 4. Forty candidate configurations were reproducibly sampled for each architecture using a fixed search seed of 2026. GCN and GraphSAGE share the same sampled common configurations, whereas the GAT configurations are sampled from the expanded architecture-specific search space.

Each configuration was evaluated using five predefined training–validation splits generated separately within each campus, with approximately 70% of the spatial units assigned to training and 30% assigned to validation in each split. Training was repeated for every split using three fixed random seeds (42, 123, and 2026) to reduce the variability associated with the stochastic initialization. The models were trained using the MAE loss with a maximum of 500 epochs and an early-stopping patience of 50. Regularization and shallow GNN architectures have been used to reduce overfitting and limit oversmoothing (68).

For the model evaluation, the seed-level results were first averaged within each campus–split combination. The performance was assessed using RMSE, MAE, and *R*^2^. The overall validation RMSE was used as the primary criterion for hyperparameter and architecture selection, with RMSE variability, MAE, and *R*^2^ as secondary ranking criteria. As each campus contributed five predefined splits, the two campuses contributed equally to the overall model-selection results. The best-performing hyperparameter configuration was first identified within each architecture, after which GCN, GraphSAGE, and GAT were compared separately for Flow and Dwell to determine the final architecture for each target.

Because the available samples were limited, validation subsets were used for both early stopping and architecture/hyperparameter selection. Consequently, the resulting validation performance may be subject to some optimistic bias and was, therefore, interpreted primarily for relative comparison among candidate architectures and hyperparameter configurations, rather than as a fully independent estimate of generalization performance.

#### 2.3.4 Benchmark design and robustness evaluation

After the GNN architecture and hyperparameter configuration were selected separately for Flow and Dwell, the selected models were compared with three deterministic benchmark models using the same five predefined data splits generated separately within each campus. The benchmark set included a mean baseline, ordinary least-squares (OLS) regression, and a spatial lag model (SLM). The mean baseline was assigned as the mean target value of the training nodes to all validation nodes. To avoid rank-deficiency issues caused by predictors with no variation in small training subsets, OLS and SLM were fitted using a common six-predictor subset across all splits. BRG, MI, and SFD were excluded because they showed no variation in at least one campus or training split, which would otherwise cause rank deficiency; therefore, the remaining predictors were used consistently across all baseline models.

For the SLM, the campus-specific adjacency structure used in the GNN analysis was retained and row-standardized to construct the spatial-weight matrix. Model estimation used only the training-node responses, whereas the full campus graph structure was retained throughout the fitting process. GWR was not included because the small number of spatial units within each campus resulted in limited effective local sample sizes and potentially unstable local coefficient estimates.

All benchmark models and selected GNNs were evaluated on identical validation nodes using RMSE, MAE, and *R*^2^. The deterministic benchmark models produce one result for each split, whereas the selected GNN results are averaged across three fixed random seeds for each split. Performance was then summarized across the five splits within each campus using mean ± standard deviation and 95% confidence intervals. For the overall target-level comparison, the two campuses were assigned equal weights, yielding ten campus–split observations for each model and target.

#### 2.3.5 Feature perturbation and node-level sensitivity analysis

After model training, a permutation-based feature perturbation approach was employed to evaluate the sensitivity of model outputs to individual built-environment features. Sensitivity analysis was performed using 60 fixed final GNN checkpoints defined as campus × split × target × seed (2 × 5 × 2 × 3) without retraining the models. Within each trained model, nine built-environment features were separately perturbed. For each feature, the values were independently permuted 30 times across all spatial units, whereas the remaining eight features, graph structure, and trained model parameters were fixed. The modified feature matrix was then passed through the trained GNN to obtain the updated node-level predictions. For each permutation, the node-level sensitivity is quantified as the absolute difference between the original and perturbed predictions, as shown in Equation 4:

$$\Delta y_{i,k}=|f_{\theta }({x_{i}}^{\left(k,\;r\right)})-f_{\theta }(x_{i})|$$ (4)

where $f_{\theta }(\cdot )$ denotes the trained GNN model, $x_{i}\in {\mathbb{R}}^{9}$ is the original node-feature vector of node i , and $x_{i}^{\left(k,r\right)}$ represents the corresponding feature vector of node i after the k−th feature was permuted across all spatial units in the r−th run.

To improve the robustness across model-training variability, these estimates were further aggregated hierarchically using medians across random seeds and data splits, yielding an aggregated node-level sensitivity index for each feature. The node-level sensitivity values were further aggregated at two higher spatial levels. First, the nodes were grouped according to the predefined Flow–Dwell quadrant classification within each campus, and their sensitivity values were aggregated to obtain quadrant-level sensitivity rankings. Second, the sensitivity values across all nodes within each campus were aggregated to derive campus-level global sensitivity rankings. These sensitivity measures and rankings represent the relative responsiveness of the model outputs to individual feature perturbations within the same model; therefore, absolute sensitivity magnitudes were not directly compared across different targets or campuses.

#### 2.3.6 Data processing for design translation

Robust normalization was applied separately to the built-environment features within each campus to provide a split-independent representation of the existing built environment conditions for design translation. This transformation was based on the median and median absolute deviation (MAD), as shown in Equation 5:

$$\tilde{x_{i,k}}=\frac{x_{i,k}-\text{median}(X_{k})}{\mathrm{MAD}(X_{k})}$$ (5)

where $x_{i,k}$ denotes the value of feature k for spatial unit i , $X_{k}=\{x_{i,k}\}$ denotes the set of values for feature k across all spatial units within the corresponding campus, and MAD(·) represents the median absolute deviation. The normalized values were used to identify the relative strengths and weaknesses of each spatial unit and were subsequently interpreted together with the sensitivity results to determine potential optimization priorities.

## 3 Results

The final analytical sample comprised 20 spatial units at WSC and 22 at GZIC, including six bridge units at GZIC. The final analytical sample comprised 42 spatial units. The spatial delineation and corresponding adjacency-based graph structures are illustrated in Figures 3, 8, respectively.

**Figure 8:**
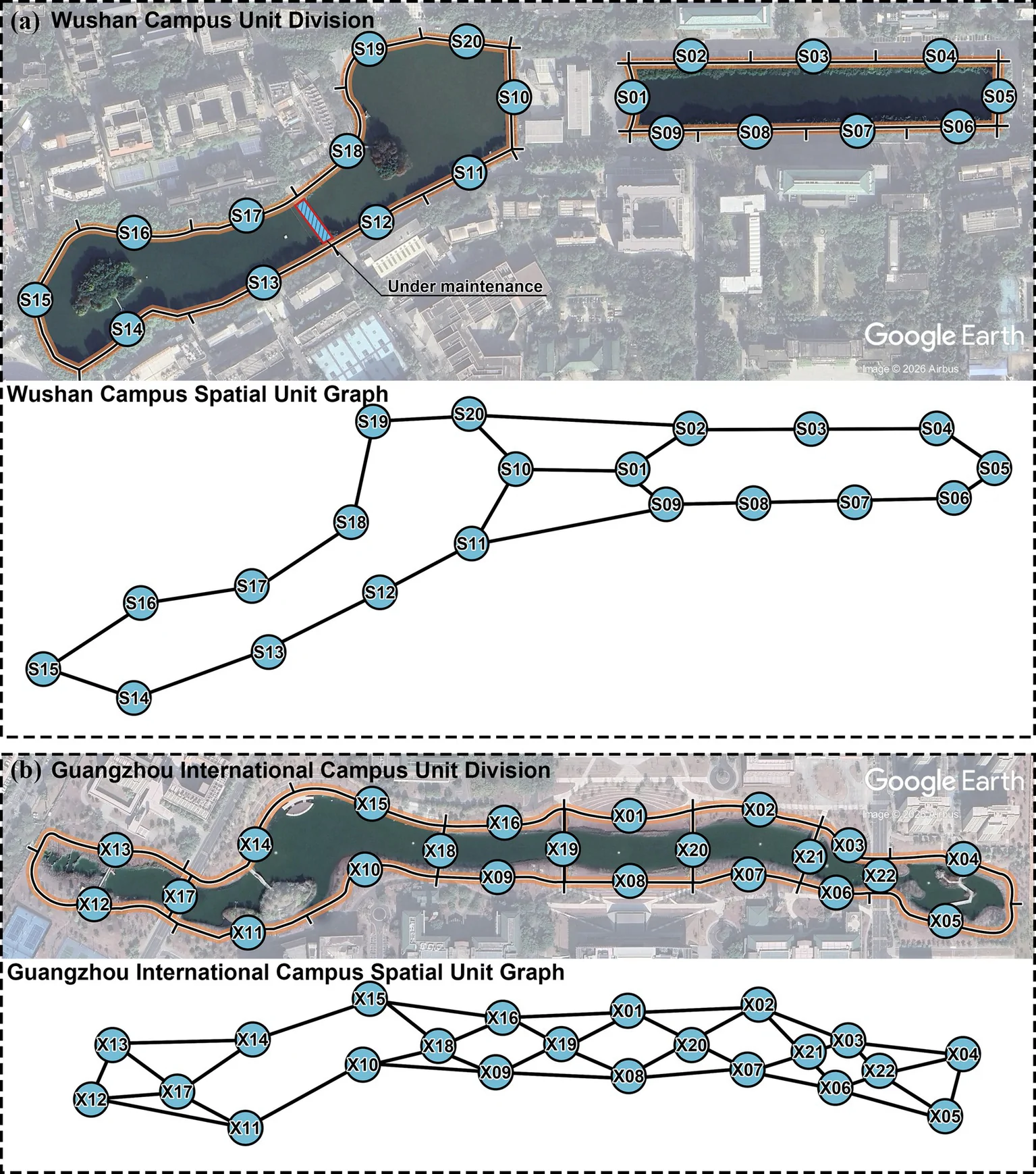
Graph structure of spatial unit adjacency for **(a)** WSC and **(b)** GZIC.

### 3.1 Spatial unit classification in Flow–Dwell quadrants

Overall, the distribution of spatial units across the four Flow–Dwell quadrants was broadly similar between the two campuses (Figure 9). At WSC, Q1–Q4 contained 5, 5, 8, and 2 spatial units, respectively, whereas at GZIC the corresponding numbers were 8, 3, 9, and 2. Thus, some quadrants, particularly Q4 at both campuses and Q2 at GZIC, contained relatively few spatial units. Therefore, the subsequent quadrant-level sensitivity results for these contexts were interpreted cautiously. Several units exhibit comparatively high indicator values. At WSC, S14 showed a relatively high Flow, whereas S17 showed a relatively high Dwell. At GZIC, X14 and X18 exhibited comparatively high Flow and Dwell, respectively. These units were retained in subsequent analyses because they represented observed spatial-use patterns rather than invalid observations.

**Figure 9:**
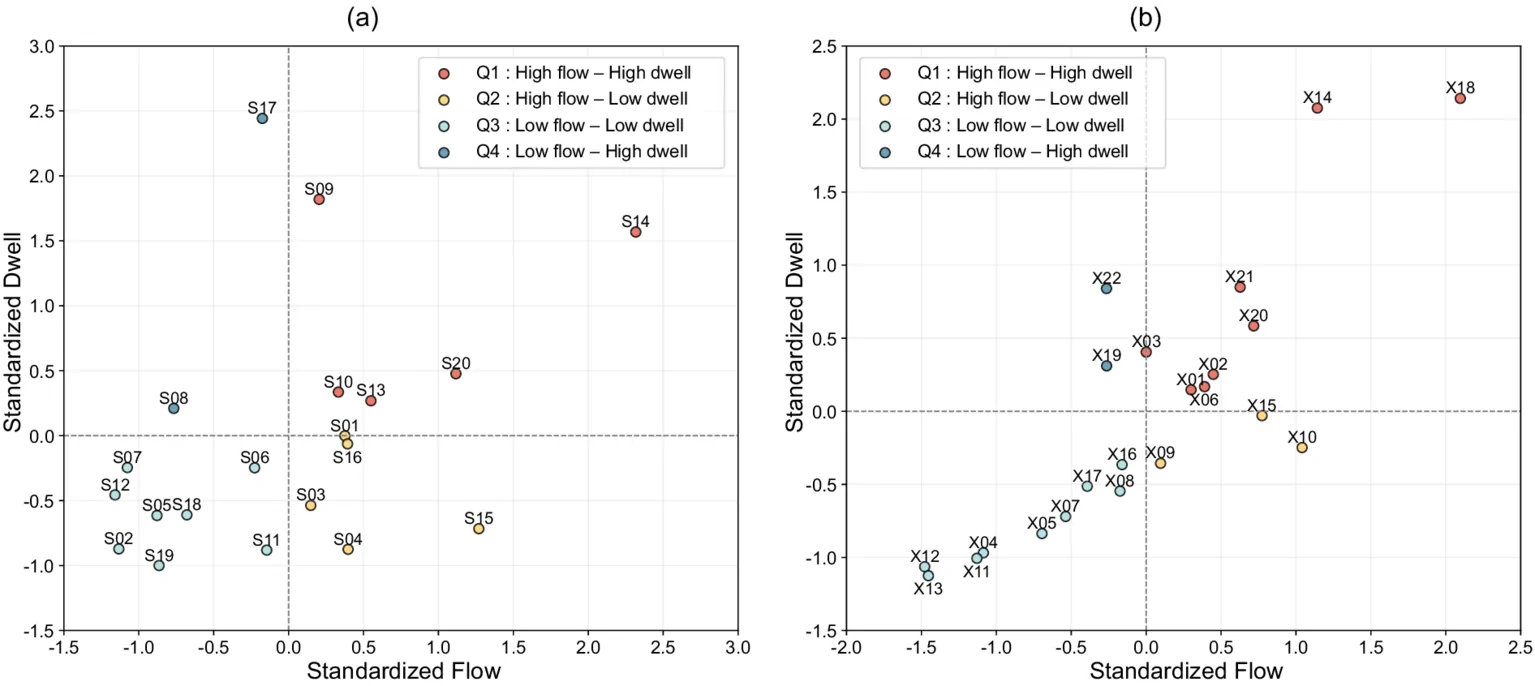
Spatial unit distribution for **(a)** WSC and **(b)** GZIC.

### 3.2 GNN architecture and hyperparameter selection results

Three GNN architectures, GCN, GraphSAGE, and GAT, were evaluated separately for Flow and Dwell using a predefined random-search procedure. Table 8 summarizes the best-performing hyperparameter configurations identified for each architecture and target, along with the corresponding overall RMSE, MAE, and *R*^2^.

**Table 8:** Best-performing hyperparameter configurations and corresponding overall performance for each GNN architecture and target; selected architectures are shown in bold.

Target	Model	Best config	Overall RMSE	Overall MAE	Overall *R*^2^
**Flow**	**GCN**	**hidden_dim = 64 num_layers = 1** **dropout = 0.3 lr = 0.005** **weight_decay = 5e-4**	**0.5387**	**0.4383**	**0.4819**
Flow	GraphSAGE	hidden_dim = 64 num_layers = 3 dropout = 0.5 lr = 0.005 weight_decay = 5e-4	0.6412	0.5231	0.3156
Flow	GAT	hidden_dim = 64 num_layers = 3 dropout = 0.5 lr = 0.005 weight_decay = 5e-4 heads = 4	0.5727	0.4697	0.4407
Dwell	GCN	hidden_dim = 32 num_layers = 1 dropout = 0.5 lr = 0.005 weight_decay = 5e-4	0.6948	0.5504	−0.0158
**Dwell**	**GraphSAGE**	**hidden_dim = 8 num_layers = 2** **dropout = 0.2 lr = 0.005** **weight_decay = 5e-4**	**0.6639**	**0.5320**	**0.0424**
Dwell	GAT	hidden_dim = 64 num_layers = 1 dropout = 0.2 lr = 0.005 weight_decay = 0 heads = 2	0.6704	0.5217	0.0175

For Flow, GCN achieved the lowest overall RMSE (0.5387), with an MAE of 0.4383 and *R*^2^ of 0.4819, and was therefore selected as the final architecture. GAT ranked second in terms of RMSE (0.5727), followed by GraphSAGE (0.6412). For Dwell, GraphSAGE achieved the lowest overall RMSE (0.6639), with an MAE of 0.5320 and an *R*^2^ of 0.0424, and was selected as the final architecture. Although GAT yielded a slightly lower MAE (0.5217), GraphSAGE was retained because the overall validation RMSE was the predefined primary selection criterion.

Overall, the candidate architectures showed a better model fit for Flow than for Dwell, particularly in terms of the explained variance. The selected GCN configurations for Flow and GraphSAGE configurations for Dwell were subsequently fixed for benchmark comparison and feature perturbation analyses.

### 3.3 Benchmark comparison and robustness results

The selected GNN models were compared with the Mean, OLS, and SLM benchmarks using the same predefined data splits (Table 9). Across the ten campus–split combinations, the selected GNN achieved the lowest overall RMSE and MAE and the highest *R*^2^ for both targets. For Flow, the mean baseline was the best-performing conventional benchmark, with an RMSE of 0.845 ± 0.248, whereas the selected GCN achieved 0.539 ± 0.173. This corresponded to an RMSE reduction of 36.2%. GCN also reduced MAE from 0.725 to 0.438 and increased mean *R*^2^ from −0.207 to 0.482.

**Table 9:** Predictive performance of benchmark models and selected GNN models for Flow and Dwell across ten campus–split combinations, reported as mean ± SD with 95% confidence intervals.

Target	Model	RMSE	MAE	*R* ^2^
Flow	Mean	0.845 ± 0.248 [0.668, 1.023]	0.725 ± 0.200 [0.582, 0.868]	−0.207 ± 0.345 [−0.455, 0.040]
	OLS	0.878 ± 0.319 [0.650, 1.106]	0.729 ± 0.217 [0.574, 0.885]	−0.393 ± 0.672 [−0.874, 0.088]
	SLM	1.134 ± 0.454 [0.809, 1.458]	0.896 ± 0.334 [0.657, 1.135]	−1.878 ± 2.671 [−3.789, 0.033]
	**GCN**	**0.539 ± 0.173** **[0.415, 0.662]**	**0.438 ± 0.143** **[0.336, 0.541]**	**0.482 ± 0.267** **[0.291, 0.673]**
Dwell	Mean	0.772 ± 0.307 [0.552, 0.991]	0.620 ± 0.259 [0.434, 0.805]	−0.278 ± 0.375 [−0.547, −0.010]
	OLS	0.977 ± 0.288 [0.772, 1.183]	0.845 ± 0.270 [0.652, 1.039]	−3.289 ± 6.647 [−8.044, 1.466]
	SLM	1.465 ± 0.738 [0.937, 1.993]	1.146 ± 0.486 [0.798, 1.494]	−7.063 ± 8.826 [−13.376, −0.750]
	**GraphSAGE**	**0.664 ± 0.258** **[0.479, 0.848]**	**0.532 ± 0.196** **[0.392, 0.672]**	**0.042 ± 0.227** **[−0.120, 0.205]**

Selected GNN results were averaged across three random seeds within each split; the best-performing model for each target is shown in bold.

For Dwell, the mean baseline again showed the lowest overall RMSE among the conventional benchmarks (0.772 ± 0.307), while the selected GraphSAGE achieved 0.664 ± 0.258, corresponding to an RMSE reduction of 14.0%. MAE decreased from 0.620 to 0.532, while mean *R*^2^ increased from −0.278 to 0.042. Thus, the RMSE reduction relative to the best-performing conventional baseline was larger for Flow than for Dwell, while Dwell retained a comparatively weak explained variance.

The selected GNNs also showed lower variability in RMSE across the ten campus–split combinations than OLS and SLM for both targets. The corresponding standard deviations and 95% confidence intervals are reported in Table 9.

### 3.4 Multi-level feature sensitivity results

For existing waterfront pedestrian spaces in campus environments, some spatial features are difficult to modify through short-term interventions. Therefore, the features were categorized into structural and design-related environmental features based on their intervention potential. AIL and BRG were treated as structural features, whereas the remaining seven features were considered more readily adjustable and were therefore prioritized in the subsequent design translation. Because the Flow and Dwell models were trained independently, sensitivity results were interpreted primarily through relative rankings within each target rather than direct comparison of absolute sensitivity magnitudes.

#### 3.4.1 Campus-level sensitivity ranking

Figure 10 compares the campus-level sensitivity rankings for the Flow and Dwell models at WSC and GZIC. For Flow, MI, WVR, and SFD ranked highest at WSC, with sensitivity values of 0.160, 0.152, and 0.135, respectively. At GZIC, WVR remained highly ranked and placed first (0.135), followed by SL (0.073) and WW (0.062). The largest cross-campus ranking shifts were observed for MI and SFD; they ranked first and third at WSC but decreased to seventh and ninth at GZIC, with SFD reaching zero. Conversely, SL and WW were ranked higher at GZIC than at WSC. For Dwell, SFD and MI ranked first and second at WSC (0.037 and 0.036, respectively), whereas they occupied the two lowest positions at GZIC. GVR showed the opposite pattern, increasing from seventh at WSC (0.028) to first at GZIC (0.083). At WSC, the sensitivity values for all features except BRG were relatively closely distributed, ranging from 0.027 to 0.037, whereas GVR was more clearly separated as the top-ranked feature at GZIC.

**Figure 10:**
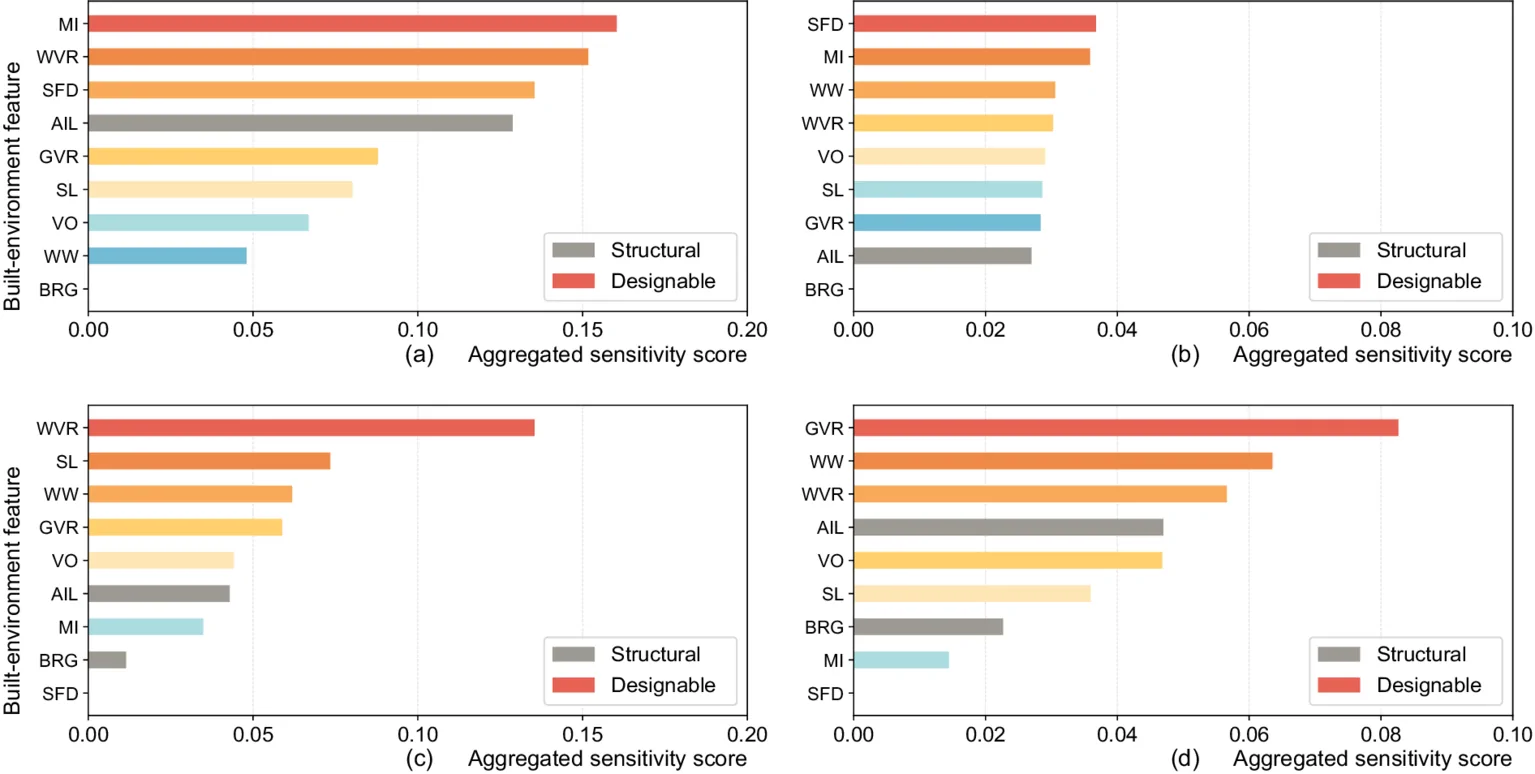
Campus-level sensitivity rankings of built-environment features for the Flow and Dwell models: **(a)** Flow at WSC; **(b)** Dwell at WSC; **(c)** Flow at GZIC; and **(d)** Dwell at GZIC. Design-related features are represented by colored bars; structural features are shown in gray.

#### 3.4.2 Quadrant-level sensitivity ranking

Figures 11–14 present the quadrant-level sensitivity rankings of built-environment features for the Flow and Dwell models at the two campuses. Because WSC Q4 (*n* = 2), GZIC Q2 (*n* = 3), and GZIC Q4 (*n* = 2) contained relatively few spatial units, the patterns observed in these quadrants were interpreted cautiously.

(1) WSC

**Figure 11:**
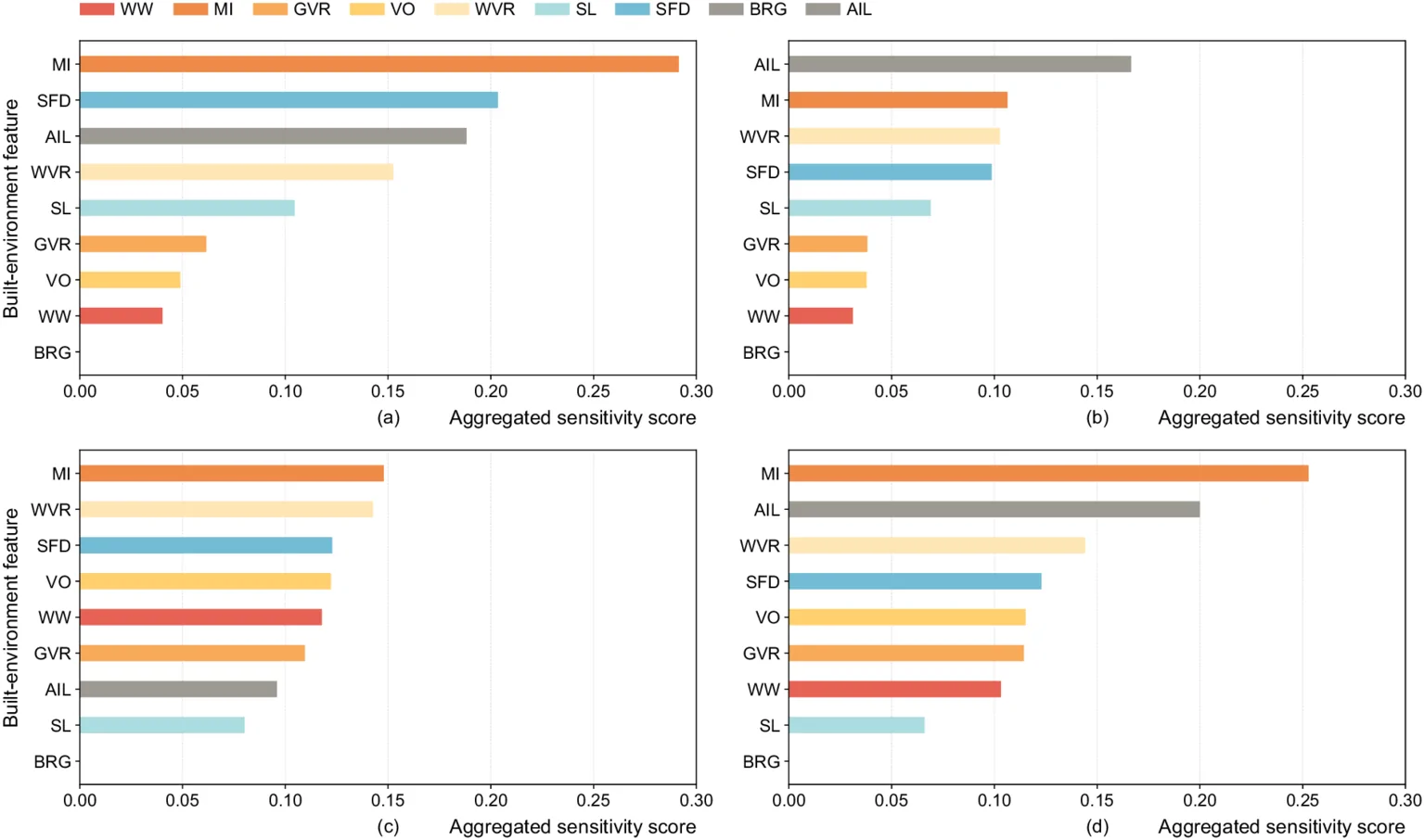
Quadrant-level sensitivity rankings of built-environment features for the Flow model at WSC: **(a)** Q1, **(b)** Q2, **(c)** Q3, and **(d)** Q4. Design-related features are represented by colored bars; structural features are shown in gray.

**Figure 12:**
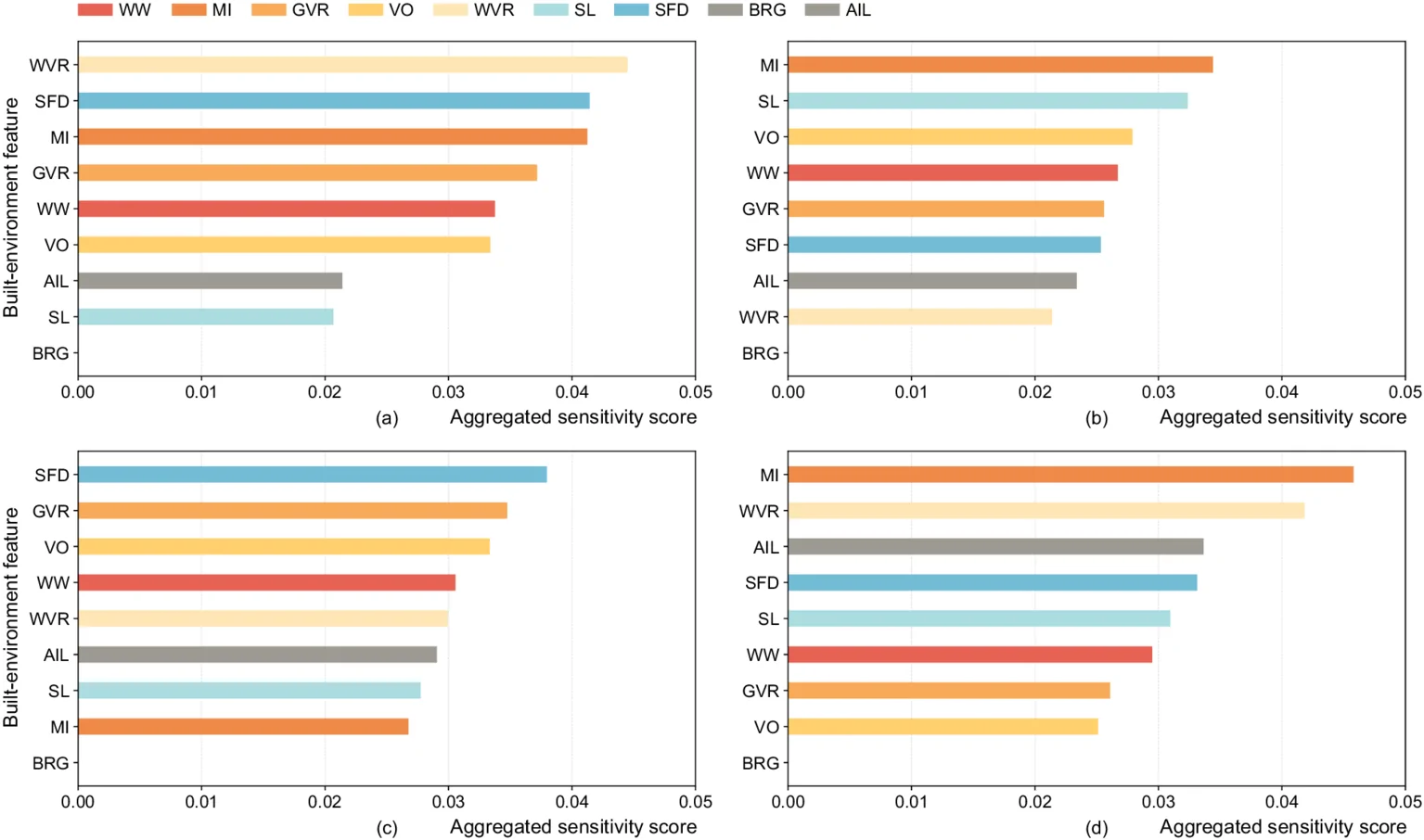
Quadrant-level sensitivity rankings of built-environment features for the Dwell model at WSC: **(a)** Q1, **(b)** Q2, **(c)** Q3, and **(d)** Q4. Design-related features are represented by colored bars; structural features are shown in gray.

**Figure 13:**
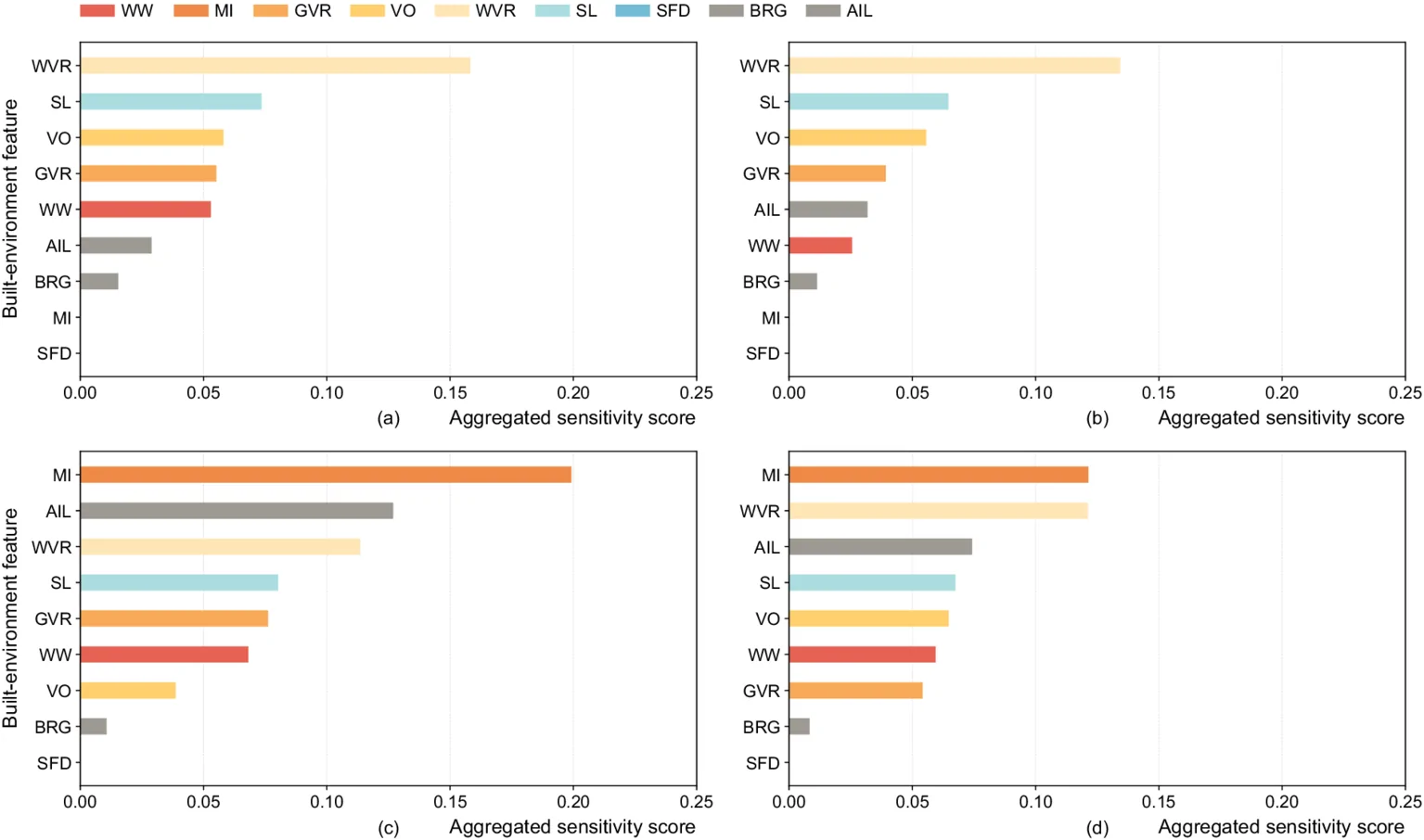
Quadrant-level sensitivity rankings of built-environment features for the Flow model at GZIC: **(a)** Q1, **(b)** Q2, **(c)** Q3, and **(d)** Q4. Design-related features are represented by colored bars; structural features are shown in gray.

**Figure 14:**
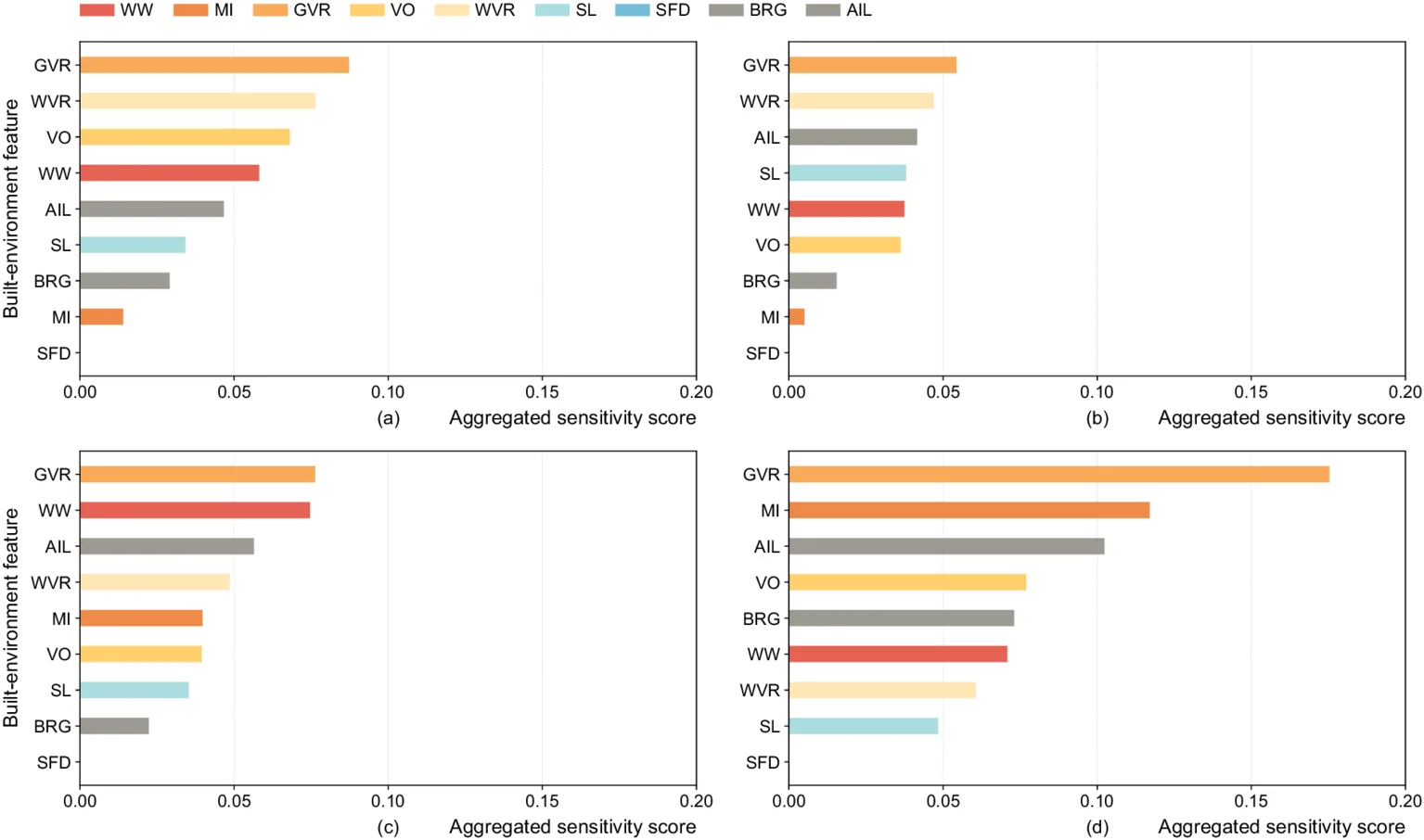
Quadrant-level sensitivity rankings of built-environment features for the Dwell model at GZIC: **(a)** Q1, **(b)** Q2, **(c)** Q3, and **(d)** Q4. Design-related features are represented by colored bars; structural features are shown in gray.

For Flow, MI remained within the top two positions across all four quadrants, ranking first in Q1, Q3, and Q4 with sensitivity values of 0.291, 0.148, and 0.253, respectively, and second in Q2 (0.106; Figure 11). SFD and WVR also remained within the top four quadrants. AIL showed the clearest ranking shift, ranking first in Q2 (0.167) but decreased to seventh in Q3 (0.096); it ranked third in Q1 and second in Q4.

For Dwell, the highest-ranked feature varied more clearly among the quadrants (Figure 12). WVR ranked first in Q1 (0.045) but decreased to eighth in Q2 (0.021) and fifth in Q3 (0.030) before returning to second in Q4 (0.042). MI ranked first in Q2 and Q4, but decreased to eighth in Q3, where SFD ranked first (0.038). Thus, the WSC Flow rankings retained a relatively stable upper-ranked group centered on MI, SFD, and WVR, whereas the leading positions for Dwell shifted among WVR, MI, and SFD. Nevertheless, the Q4 rankings should be treated as descriptive, because they are derived from only two spatial units. The BRG remained at zero across all WSC quadrants for both models.

(2) GZIC

For Flow, WVR ranked first in Q1 and Q2, with sensitivity values of 0.158 and 0.134, respectively, but ranked third in Q3 and second in Q4 (Figure 13). The most distinct ranking change was observed for MI; it had zero sensitivity in Q1 and Q2 but ranked first in Q3 (0.199) and Q4 (0.121). The AIL showed a similar upward shift, moving from the sixth and fifth positions in Q1 and Q2 to the second and third positions in Q3 and Q4. These changes produced a clear contrast between the WVR-led rankings in Q1–Q2 and the MI-led rankings in Q3–Q4, although the Q2 and Q4 patterns were based on small samples.

For Dwell, GVR remained the top-ranked feature across all four quadrants, ranging from 0.054 in Q2 to 0.175 in Q4 (Figure 14). WVR ranked second in Q1 and Q2, but decreased to fourth in Q3 and seventh in Q4. Conversely, MI ranked eighth in Q1 and Q2, increased to fifth in Q3, and ranked second in Q4; the latter shift should be interpreted cautiously, given the Q4 sample size. SFD remained zero throughout the GZIC quadrants. Overall, the contrast between the quadrant-specific ranking profiles was clearest for Flow at GZIC, whereas the Dwell rankings consistently retained GVR in the leading position.

## 4 Discussion

The modeling results showed clear differences between the two vitality targets, with Flow being more accurately predicted than Dwell, and the selected GNNs outperforming the tested conventional baselines under the current setup. Perturbation analysis further revealed campus- and quadrant-specific variations in feature-sensitivity patterns. These findings suggest that the spatial associations captured by the models are context-dependent and should be interpreted as model-based associations rather than causal effects. The following sections discuss these patterns at campus, quadrant, and unit levels.

### 4.1 Campus-level spatial response patterns

Campus-level sensitivity rankings show clear differences between WSC and GZIC, with only a few shared patterns. WVR ranked high for Flow at both campuses, whereas MI, SFD, GVR, SL, and WW showed larger differences in their relative rankings. This suggests that the sensitivity patterns varied with campus context rather than following the same ranking structure across campuses.

The clearest shared pattern was the highest ranking of WVR for Flow on both campuses. This was observed despite clear differences in the waterfront form: WSC has a more discontinuous water system with a direct pedestrian–water interface, whereas GZIC has a more continuous water system with a buffered interface. This shows that water visibility was model-sensitive at both campuses, although any design response depended on the local waterfront layout. BRG was not discussed further because WSC had no bridge units, and only six were present at GZIC.

The contrasting MI and SFD rankings may be related to differences in traffic organization and waterfront facilities. At WSC, mixed pedestrian–vehicle use remains common along several waterfront sections despite formal route separation. This may explain why MI ranked first in the Flow model and second in the Dwell model. At GZIC, pedestrian–vehicle separation was more consistently maintained, with mixed use occurring mainly on the two bridges, which may be related to the lower ranking of MI in both models. SFD showed a similar cross-campus contrast. WSC provides numerous waterfront seats and SFD ranked third for Flow and first for Dwell. GZIC has no waterside seating, which corresponds to zero SFD sensitivity in both models. These patterns reflect differences in facility availability and spatial variation, rather than the uniform effect of seating on pedestrian activity.

The rankings of GVR, SL, and WW may also be related to differences in landscape and walkway conditions. WSC has mature vegetation and dense tree shade, whereas GZIC has younger trees, less canopy cover, and more uneven shading. These conditions may make local differences in GVR more salient in the GZIC Dwell model, in which GVR ranked first compared to seventh at WSC. For Flow, SL and WW ranked second and third at GZIC, but sixth and eighth at WSC, respectively. Although GZIC contains only two roofed bridges, the contrast between their complete overhead cover and limited tree shade elsewhere may partly explain the higher SL ranking in its Flow model. WW may also be more prominent in the GZIC Flow model because its walkways are generally wider than those at WSC, although the ranking does not indicate that greater width necessarily produces higher Flow.

### 4.2 Quadrant-level spatial response patterns

Quadrant-level sensitivity patterns were interpreted in relation to the four combinations of Flow and Dwell, with the rankings considered separately for each model. These profiles describe how model responsiveness varies across activity contexts, rather than establishing causal effects on pedestrian behavior. Because several quadrants contained few spatial units, particularly WSC Q4, GZIC Q2, and GZIC Q4, the Top-5 rankings are treated as descriptive summaries rather than robust statistical evidence.

#### 4.2.1 Q1: High Flow–High Dwell

Q1 included spaces with both high movement and sustained presence. The main question was whether the Flow and Dwell models were sensitive to some of the same spatial features in mixed-use spaces (Table 10). At WSC, MI, SFD, and WVR were among the Top-5 features in both models. At GZIC, WVR, GVR, VO, and WW were the Top-5 of both models. Overall, several spatial dimensions were prominent in both the Flow and Dwell models.

**Table 10:** Comparison of the top five features by aggregated sensitivity in Q1 between WSC and GZIC; rankings are presented as descriptive summaries rather than inferential statistical evidence.

Campus	Target	Top 1	Top 2	Top 3	Top 4	Top 5
WSC	Flow	MI	SFD	AIL	WVR	SL
	Dwell	WVR	SFD	MI	GVR	WW
GZIC	Flow	WVR	SL	VO	GVR	WW
	Dwell	GVR	WVR	VO	WW	AIL

The specific feature combinations differed between the two campuses. At WSC, the ranking pattern may be understood in relation to mixed pedestrian–vehicle use, waterfront seating, and direct views of water. At GZIC, the higher rankings of landscape, openness, and walkway features were consistent with a continuous water system, buffered waterfront interface, and wider paths. WVR was the most shared feature between the campuses, suggesting that water visibility is a recurring model-sensitive dimension in High Flow–High Dwell spaces. These rankings do not explain why the units belong to Q1. They identified the spatial dimensions that may need to be considered when supporting both movement and sustained presence.

#### 4.2.2 Q2: High Flow–Low Dwell

Q2 includes movement-oriented spaces with high Flow but relatively low Dwell. The main question is whether the features prominent in the Flow model differed from those to which the Dwell model remained sensitive (Table 11). At WSC, AIL and MI ranked highest for Flow, while MI and SL ranked highest for Dwell. At GZIC, WVR and SL ranked highest for Flow, while GVR and WVR ranked highest for Dwell.

**Table 11:** Comparison of the top five features by aggregated sensitivity in Q2 between WSC and GZIC; rankings are descriptive, and the GZIC results should be interpreted cautiously because Q2 contained only three spatial units.

Campus	Target	Top 1	Top 2	Top 3	Top 4	Top 5
WSC	Flow	AIL	MI	WVR	SFD	SL
	Dwell	MI	SL	VO	WW	GVR
GZIC	Flow	WVR	SL	VO	GVR	AIL
	Dwell	GVR	WVR	AIL	SL	WW

Only a few highly ranked features were shared between the two models. The high ranking in the Dwell model does not explain why Dwell was low. Instead, it identified features that may require closer examination when considering how movement-oriented spaces can accommodate stays. The WSC pattern may be related to the route structure and mixed pedestrian–vehicle conditions. The GZIC pattern may be related to a continuous water view and uneven shading. However, GZIC Q2 contains only three units; therefore, these interpretations are tentative and need to be verified at the individual-unit level.

#### 4.2.3 Q3: Low Flow–Low Dwell

Q3 included spaces with relatively low levels of both Flow and Dwell. These spaces should not be treated automatically as uniformly deficient. Instead, the sensitivity results showed which local spatial features remained prominent in the models in the low-activity context (Table 12). At WSC, SFD, WVR, VO, and WW appeared in the Top-5 of both models. At GZIC, MI, AIL, WVR, and GVR were the Top-5 of both models. Similarly, low activity levels were associated with different sensitivity patterns across campuses.

**Table 12:** Comparison of the top five features by aggregated sensitivity in Q3 between WSC and GZIC; rankings are presented as descriptive summaries rather than inferential statistical evidence.

Campus	Target	Top 1	Top 2	Top 3	Top 4	Top 5
WSC	Flow	MI	WVR	SFD	VO	WW
	Dwell	SFD	GVR	VO	WW	WVR
GZIC	Flow	MI	AIL	WVR	SL	GVR
	Dwell	GVR	WW	AIL	WVR	MI

This pattern was particularly clear for MI at GZIC. MI ranked relatively low in campus-level Flow results but ranked first in the Q3 Flow model. This indicates that campus-level aggregation may hide sensitivity patterns concentrated in a particular activity context. The Q3 rankings did not identify the causes of low activity. Instead, they show the spatial dimensions that remain relevant to model responses in different types of low-activity units. Therefore, they provide context for a more detailed analysis at the individual-unit level.

#### 4.2.4 Q4: Low Flow–High Dwell

Q4 included stay-oriented spaces with a highly sustained presence but relatively low movement. The main question is which spatial features are prominent in the Dwell model and whether these features also appear in the Flow ranking for the same units. In the observed WSC units (Table 13), WVR and SFD ranked highly for Dwell and appeared in the Flow Top-5. This is consistent with WSC’s direct waterfront interface and waterside seating. MI and AIL also ranked highly in both models. This indicates that the sensitivity patterns of these stay-oriented units were not limited to comfort-related features or stay facilities.

**Table 13:** Comparison of the top five features by aggregated sensitivity in Q4 between WSC and GZIC; rankings are exploratory because both WSC and GZIC Q4 contained only two spatial units.

Campus	Target	Top 1	Top 2	Top 3	Top 4	Top 5
WSC	Flow	MI	AIL	WVR	SFD	VO
	Dwell	MI	WVR	AIL	SFD	SL
GZIC	Flow	MI	WVR	AIL	SL	VO
	Dwell	GVR	MI	AIL	VO	BRG

At GZIC, GVR ranked first for Dwell, whereas MI, AIL, and VO appeared in the Top-5 of both models. Therefore, the observed GZIC units exhibited different sensitivity patterns. Greenery and spatial structures were more prominent than stay facilities and were absent along the waterfront. This comparison suggests that a high Dwell can occur alongside different sensitivity patterns at different campuses rather than being associated with one common set of features. However, each campus contained only two Q4 units. Therefore, these findings should be treated as exploratory and used only to guide further analyses at the individual-unit level.

Across the four quadrants, the degree of overlap between the Flow and Dwell sensitivity rankings varied by activity context, whereas similarly classified units did not necessarily exhibit uniform sensitivity patterns across the campuses. These quadrant-level results provide the behavioral context for subsequent unit-level analysis but should not be treated as direct design instructions.

### 4.3 Unit-level spatial response patterns and design translation

Unit-level sensitivity rankings, Flow–Dwell classifications, and current feature levels were combined to identify possible priorities for spatial improvement. This approach links each unit’s behavioral context with the environmental features to which its Flow and Dwell models are the most responsive.

#### 4.3.1 Unit-level sensitivity-based translation

The unit-level sensitivity indicates how strongly a model responds when a feature is perturbed. This does not show whether increasing this feature will necessarily improve Flow or Dwell. Therefore, sensitivity should be interpreted together with the current feature level, the desirability of its direction, and whether the feature can be modified through design.

For most design-related features, a high sensitivity ranking combined with a below-median current level identified possible priorities for further design evaluation. Instead, a highly sensitive feature that is already well provided may need to be maintained. MI requires the opposite interpretation because a higher value represents greater motor interference, and therefore, a less desirable condition. Features with low sensitivity are generally given a lower priority in model-based translations, even when their current levels are low. Based on these principles, a “sensitivity–current condition” framework was used to distinguish between features that should be maintained, features that may be improved, and features that require further site-level examination. Because the sensitivity values describe the response magnitude rather than the direction, all proposed interventions should be treated as design hypotheses rather than as predicted behavioral effects.

#### 4.3.2 Unit-level optimization examples

Four units were retained as illustrative examples: two Q1 composite high-activity spaces from different campuses (S09 and X01), one Q2 movement-oriented space (S15), and one Q3 low-activity space (X07). Their feature levels were robust-standardized within each campus (Figure 15). Therefore, positive and negative values represent conditions above and below the corresponding campus median rather than the absolute differences between campuses. Q4 was not included because only two units were available on each campus, making unit selection less reliable.

(1) Q1 Composite High-activity Spaces (S09, X01)

**Figure 15:**
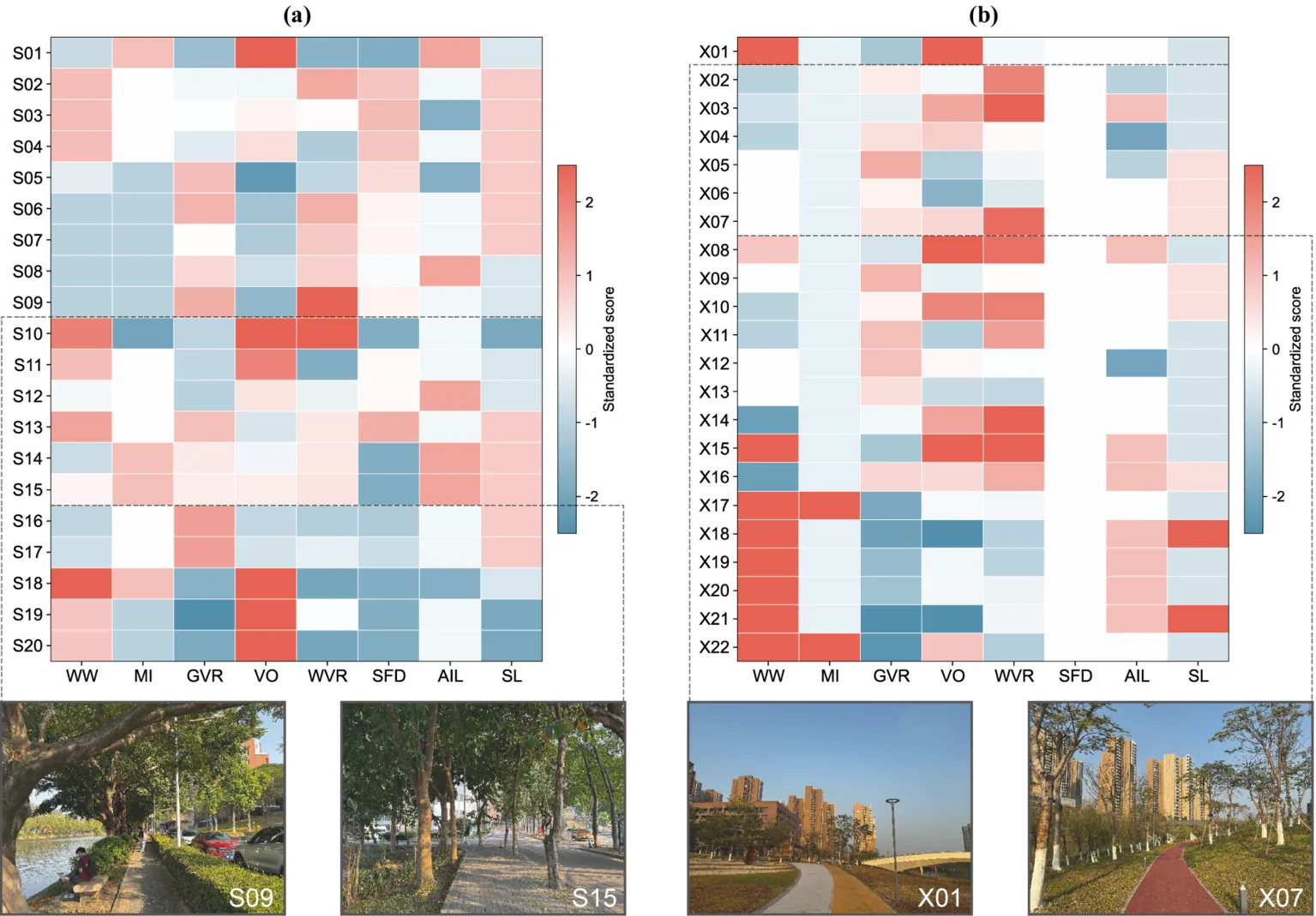
Heat map showing the standardized levels of spatial features on the two campuses: **(a)** WSC and **(b)** GZIC. For MI, warmer colors correspond to stronger motor interference and thus indicate less desirable conditions.

For Q1 units, design translation focuses on maintaining the spatial conditions associated with their existing High Flow–High Dwell profile, while identifying features that may warrant further evaluation. At S09, SFD, MI, and WVR ranked highly in the Flow sensitivity analysis, whereas GVR, SFD, WVR, and MI were prominent in the Dwell (Figure 16). Current WVR and GVR levels were above the WSC median, SFD was slightly above the median, and MI was relatively low. Therefore, these conditions should be maintained. The SL was below the campus median and showed moderate Flow sensitivity. Therefore, increasing local shade emerged as a possible priority for further evaluation. Although the WW and VO values were also below the median, their sensitivity rankings were relatively low. Therefore, adjustments to walkways or interface conditions may be explored only when supported by further site-level assessments.

At X01, WVR and WW ranked highest for Flow, whereas VO, GVR, and WVR ranked highest for Dwell. Its current WW and VO levels were well above the GZIC median and may, therefore, be treated as existing conditions for maintenance. By contrast, GVR was below the campus median and WVR was slightly below it. Therefore, refining vegetation configuration and strengthening water visibility should be explored as site-specific design directions. Additional planting should be carefully evaluated to avoid blocking the existing waterfront views.

(2) Q2 Movement-oriented Spaces (S15)

**Figure 16:**
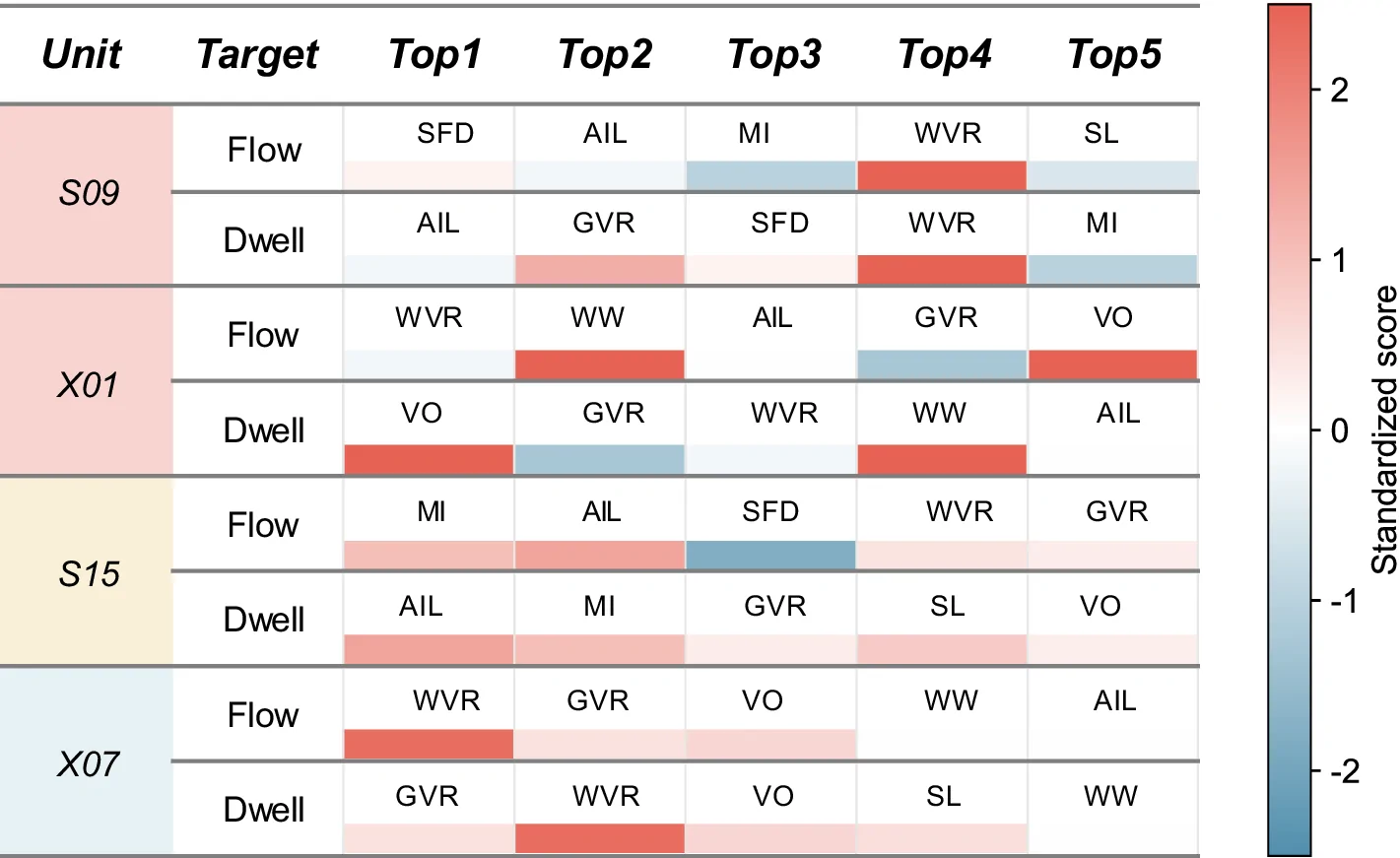
Top-five unit-level sensitivity features and campus-standardized feature levels for S09, X01, S15, and X07.

For Q2 units, the main objective is to maintain movement conditions while identifying spatial features that may better support short-term stays. At S15, MI ranked first for Flow and second for Dwell (Figure 16). The current MI level was also above the WSC median, indicating relatively strong motor interference. Therefore, reducing pedestrian–vehicle conflict emerges as a candidate priority for site-level evaluation, given its relevance to both modeled outcomes.

SFD was well below the campus median and ranked third for Flow, although it ranked lower for Dwell. Selective seating or small rest areas may therefore be explored; however, they should be located along the edge of the movement space to avoid interrupting pedestrian circulation. In contrast, SL, GVR, and WVR were above the campus median. Overall, interventions at S15 should prioritize reducing motor interference and maintaining pedestrian circulation while introducing only limited and carefully positioned rest areas.

(3) Q3 Low-activity Spaces (X07)

For the Q3 units, low Flow and low Dwell do not necessarily indicate that all measured environmental features are insufficient. At X07, WVR, GVR, and VO ranked highly in both models, while WW also entered the Top-5 rankings (Figure 16). However, WVR, GVR, VO, and SL were above the GZIC median, and WW was close to the median. Therefore, the sensitivity–current condition assessment identifies no clear combination of high sensitivity and poor existing provisions among these features.

For X07, the priority should be to maintain its water views, greenery, openness, and shading rather than apply broad physical upgrades. The low-activity profile may be related to conditions that are not fully represented by the current feature set, such as nearby functions, activity programming, temporal use, or connections beyond the immediate unit. These factors should be examined before further spatial changes are proposed. Accordingly, the current results support maintenance and further site-level examinations, rather than immediate physical interventions.

These examples demonstrate that a unit-level design translation should combine the Flow–Dwell quadrant, unit-level sensitivity, and current feature levels. This approach distinguishes between features to maintain and weaknesses requiring further attention, producing different priorities even for units in the same quadrant, such as S09 and X01. It also avoids unsupported interventions at X07, where highly sensitive features are already present at or above the campus median. Therefore, the resulting strategies are context-specific design directions that should be further assessed onsite and validated where possible.

### 4.4 Method applicability and limitations

This study developed an integrated framework that combines spatial unit delineation, behavioral data construction, graph modeling, sensitivity analysis, and design translation. It is most applicable to pedestrian environments that can be divided into relatively stable spatial units with clear spatial connections between units and provide node-level behavioral data. These conditions are found on campus waterfronts and may also occur in other continuous pedestrian networks. However, the current evidence is limited to only two university campuses. This framework can be adapted to other pedestrian environments with comparable spatial and data conditions; however, its transferability requires further empirical validation.

Several methodological conditions define the interpretation of the results. Graph representation depends on the scale and boundaries of the spatial units and on the definition of adjacency. Alternative choices may produce different representations of spatial relationships (69, 70). Sensitivity indicates the magnitude of the model response to feature perturbation but not the direction of that response or a causal effect on pedestrian behavior. Therefore, unit-level design translation should be treated as a set of design hypotheses for site assessment rather than as a prediction of intervention effects. The modeling process prioritizes interpretability and consistency rather than maximum predictive accuracy, and the stability of the results across other models and graph structures requires further testing (71).

The data provided a consistent basis for comparing the relative differences among spatial units within the observed campuses; however, the evidence reflected specific spatiotemporal and case contexts. Wi-Fi records offer a scalable proxy for aggregated movement and sustained presence rather than direct measurements of individual walking and staying. Device carriage, connection behavior, and access-point coverage may have influenced the observed records. The data were collected over a relatively short period, mainly under mild seasonal conditions, without fully capturing variations related to weather, teaching schedules, holidays, and campus events. This seasonal boundary is relevant because heat exposure may constrain outdoor activities (5, 72). Although spatial features describe shade, vegetation, waterfront visibility, facilities, and spatial form, they do not directly measure thermal exposure, acoustic quality, air pollution, or perceived restoration of users. These dimensions should be incorporated before the framework can be used to directly evaluate restorative or heat-health outcomes (9, 72). In addition, the two campuses were not designed as strictly matched cases and differences in student, staff, residential, and commuting populations were not explicitly controlled. The sample contained 42 spatial units, with only two units in WSC Q4, three in GZIC Q2, and two in GZIC Q4. Findings from these quadrants should, therefore, be interpreted as descriptive or exploratory. Bridge-related results have a similar boundary because WSC contains no bridge units and GZIC contains only six units.

Comparisons of feature levels and sensitivity values also followed specific methodological boundaries. Environmental features were robust-standardized within each campus; therefore, the standardized values describe the relative position of a unit within its own campus, rather than the absolute environmental differences between campuses. The Flow and Dwell models were trained separately and their absolute sensitivity magnitudes were not directly comparable. Therefore, the interpretation focuses on the relative rankings within each model and campus-specific sensitivity patterns.

The framework also offers planning-relevant evidence for public health research by distinguishing aggregated movement-related use from sustained presence in outdoor campus spaces. Flow provides information on spatial differences in movement-related use, whereas Dwell adds information on continued presence. Together, they help to identify environmental features and spatial units that may warrant further assessment when planning walking-supportive and activity-supportive campus environments. This contribution remains indirect because the study did not measure the duration or intensity of physical activity, sedentary behavior, mental health, restorative experience, social interaction, or health outcomes. Higher Flow does not necessarily imply more health-beneficial physical activity, and higher Dwell does not necessarily represent restorative or socially beneficial use. Therefore, the results should be interpreted as supportive spatial evidence rather than direct evidence of health effects.

Future validations could extend this framework to other campuses and pedestrian environments with comparable spatial and data conditions. Larger and more balanced quadrant samples, multiseasonal and multitemporal observations, and more detailed information on user composition would strengthen the assessment of result stability. Combining Wi-Fi data with direct behavioral observations, perceived-restorativeness, psychological surveys, wearable physiological measures, and other health-related indicators would allow a closer examination of whether flow corresponds well to particular forms of activity, restorative experience, or health-related responses (2, 9). Therefore, alternative spatial-unit scales, graph definitions, models, and external datasets must be tested.

## 5 Conclusion

A graph-based analytical framework integrating behavioral differentiation and spatial structure was developed for campus waterfront pedestrian spaces. By combining aggregated Wi-Fi probe data with multi-source spatial data, the framework characterized Flow and Dwell patterns and examined model sensitivity at the campus, behavioral-quadrant, and individual-unit levels. This multi-scale structure provides a consistent basis for interpreting spatial differences within specific behavioral and campus contexts.

The GNN architecture comparison identified the GCN for Flow and GraphSAGE for Dwell, based primarily on the validation RMSE across the two campuses and predefined data splits. Both selected architectures achieved the highest overall *R*^2^ among the GNN candidates for their respective targets. Compared to the best-performing baseline benchmark, the selected GNNs reduced the mean validation RMSE by approximately 36.2% for Flow and 14.0% for Dwell. The corresponding *R*^2^ increased from −0.207 to 0.482 for Flow and from −0.278 to 0.042 for Dwell. The performance gain was clearer for Flow, whereas the predictive performance for Dwell remained relatively modest. These results show that the selected GNNs outperformed the tested conventional baselines under the current experimental setup; however, they did not isolate the contribution of adjacency or establish causal spatial relationships. Feature perturbation was subsequently used to measure the relative responsiveness of model predictions to individual built-environment features. The resulting sensitivity values represented the response magnitude rather than the causal influence or response direction.

This analysis identified shared and campus-specific sensitivity patterns. WVR ranked highly for Flow at both campuses, whereas other feature rankings varied across campus, target, and Flow–Dwell contexts. At the unit level, combining behavioral classification, feature sensitivity, and current environmental conditions distinguished features that could be maintained, considered for adjustment, or examined further, while also showing that units within the same quadrant may require different design responses. Given the small number of units in GZIC Q2 and both Q4 groups, the corresponding quadrant patterns should be treated as descriptive, and the proposed responses are site-specific design hypotheses rather than validated intervention effects.

Overall, this study establishes a systematic pathway from multiscale model interpretation to unit-level design judgment for campus waterfront pedestrian spaces. Its main contribution lies in combining behavioral classification, spatial connections, model sensitivity, and current environmental conditions to support fine-grained and context-specific spatial evaluations. The framework also provides indirect, planning-relevant evidence for identifying walking-supportive and activity-supportive campus environments, although it does not establish direct behavioral or health effects. Based on evidence from the two campuses, the framework should not be generalized beyond pedestrian environments with comparable spatial structures and node-level data without further validation across larger samples, additional sites, and different temporal and environmental conditions.

## Data Availability

The original contributions presented in the study are included in the article, and further inquiries can be directed to the corresponding authors.

## References

[1] Akkurt E Akbulut MT. Interaction of in-between spaces in university campuses with informal learning and spatial design: a scoping review. J Asian Archit Build Eng. (2025):1–22. doi: 10.1080/13467581.2025.2570039, 37339054

[2] Guo W Wen H Liu X. Research on the psychologically restorative effects of campus common spaces from the perspective of health. Front Public Health. (2023) 11:1131180. doi: 10.3389/fpubh.2023.1131180, 37124769 PMC10135307

[3] Wen H Lin H Liu X Guo W He B-J. Analysis of the characteristics of university common spaces that affect university students’ psychological restoration. Sci Rep. (2025) 15:14146. doi: 10.1038/s41598-025-95771-8, 40269002 PMC12019356

[4] Shen Z Zhang Z Cui L Xia Z Zhang Y. Coordinated change of PM2.5 and multiple landscapes based on spatial coupling model: comparison of inland and waterfront cities. Environ Impact Assess Rev. (2023) 102:107194. doi: 10.1016/j.eiar.2023.107194

[5] Liu X He J Xiong K Liu S He B-J. Identification of factors affecting public willingness to pay for heat mitigation and adaptation: evidence from Guangzhou, China. Urban Clim. (2023) 48:101405. doi: 10.1016/j.uclim.2022.101405

[6] Guo W Li Q Wen H. The psychologically restorative effects of blue-green spaces in universities: a deep learning-based analysis of visual elements. Sustainability. (2026) 18:1780. doi: 10.3390/su18041780

[7] Ma H Kwan M-P. Seasonal variations in the restorative quality of campus landscapes: a machine learning approach using crowdsourced data. Environ Behav. (2026) 58:3–31. doi: 10.1177/00139165251381467

[8] Wang R Jiang W Lu T. Landscape characteristics of university campus in relation to aesthetic quality and recreational preference. Urban For Urban Green. (2021) 66:127389. doi: 10.1016/j.ufug.2021.127389

[9] Wen H Lin H Liu X Guo W Yao J He B-J. An assessment of the psychologically restorative effects of the environmental characteristics of university common spaces. Environ Impact Assess Rev. (2025) 110:107645. doi: 10.1016/j.eiar.2024.107645

[10] Wu Y Wang Y Wang J Li J Ding Y Ding Y . Assessing the impact of waterfront trail aesthetics on psychological restoration in urban environments: a deep learning and random forest approach. Front Public Health. (2026) 13:1757145. doi: 10.3389/fpubh.2025.1757145, 41696707 PMC12901393

[11] Ji X Fu Y Li Q Shi Z Bao K Lyu M . The impact of campus pathway landscape environment on multidimensional health benefits of university students. Buildings. (2026) 16:1454. doi: 10.3390/buildings16071454

[12] Huang G Yu Y Lyu M Sun D Dewancker B Gao W. Impact of physical features on visual walkability perception in urban commercial streets by using street-view images and deep learning. Buildings. (2025) 15:113. doi: 10.3390/buildings15010113

[13] Zhou Z Tian J Zhu Y Kang J. Impact of visual factors on soundscapes in pedestrian spaces along trunk roads. Transp Res Part D Transp Environ. (2025) 139:104583. doi: 10.1016/j.trd.2024.104583

[14] Wu T Lin D Chen Y Wu J. Integrating street view images, deep learning, and sDNA for evaluating university campus outdoor public spaces: a focus on restorative benefits and accessibility. Land. (2025) 14:610. doi: 10.3390/land14030610

[15] Bai Z Huang S Wang L Qi J. Refined understanding of street vitality: comparing the spatial effects of pedestrian vitality and cycling vitality and the spatial heterogeneity of factors. Int J Digit Earth. (2025) 18:2501769. doi: 10.1080/17538947.2025.2501769

[16] Gehl J. Life between buildings: using public space. Koch J, translator. Washington, DC: Island Press (2010). doi: 10.2307/jj.41003936

[17] Sant’Anna MV Zhou W Xu Y. Assessing appropriation of space in urban green spaces: three case studies in downtown Shanghai. Land (Basel). (2024) 13:1893. doi: 10.3390/land13111893

[18] Li Y Zhang Q-H Guo L Liu W-P He H. From behavioral characteristics to spatiotemporal structures: identifying urban active-healthy walking support types and their environmental determinants. Buildings. (2026) 16:1182. doi: 10.3390/buildings16061182

[19] Lian H Li X Zhou W Zhang J Li H. Pedestrian vitality characteristics in pedestrianized commercial streets-considering temporal, spatial, and built environment factors. Front Archit Res. (2025) 14:630–53. doi: 10.1016/j.foar.2024.10.006

[20] He K Li H Zhang H Hu Q Yu Y Qiu W. Revisiting Gehl’s urban design principles with computer vision and webcam data: associations between public space and public life. Environ Plan B Urban Anal City Sci. (2026) 53:143–62. doi: 10.1177/23998083251328771

[21] Paba-Larios MF Gutiérrez M Ramos R Cantillo V. Modeling the influence of built environment on pedestrian perceptions in Colombian cities. Transp Policy. (2025) 163:358–69. doi: 10.1016/j.tranpol.2025.01.024

[22] Yuan Y Tang F Zhou S Zhang Y Li X Wang S . Interpretable assessment of streetscape quality using street-view imagery and satellite-derived environmental indicators: evidence from Tianjin, China. Buildings. (2026) 16:1. doi: 10.3390/buildings16010001

[23] Koszowski C Wittwer R Hubrich S Gerike R. Perceived importance of context-specific built-environment factors of walking: a new perspective for prioritizing policy measures for promoting walking. Int J Sustain Transp. (2024) 18:275–90. doi: 10.1080/15568318.2023.2301372

[24] Luo P Yu B Li P Liang P. Spatially varying impacts of the built environment on physical activity from a human-scale view: using street view data. Front Environ Sci. (2022) 10:1021081. doi: 10.3389/fenvs.2022.1021081

[25] Zhou H Yuan J Zhao Y Zhang J. Geospatial variations in the influence of urban environmental attributes on positive emotions. Int J Environ Res. (2025) 19:196. doi: 10.1007/s41742-025-00835-2

[26] Dong T Tadi M Tesfaye ST. Unveiling hidden green corridors: an agent-based simulation (ABS) of urban green continuity for ecosystem services and climate resilience. Smart Cities. (2025) 8:163. doi: 10.3390/smartcities8050163

[27] Lee S Sung H Woo A. The spatial variations of relationship between built environment and pedestrian volume: focused on the 2009 Seoul pedestrian flow survey in Korea. J Asian Archit Build Eng. (2017) 16:147–54. doi: 10.3130/jaabe.16.147

[28] Wang Z Ettema D Helbich M. Objective environmental exposures correlate differently with recreational and transportation walking: a cross-sectional national study in the Netherlands. Environ Res. (2021) 194:110591. doi: 10.1016/j.envres.2020.110591, 33359675

[29] Hoogendoorn SP Bovy PHL. Pedestrian route-choice and activity scheduling theory and models. Transp Res B Methodol. (2004) 38:169–90. doi: 10.1016/S0191-2615(03)00007-9

[30] Angel A Cohen A Nelson T Plaut P. Evaluating the relationship between walking and street characteristics based on big data and machine learning analysis. Cities. (2024) 151:105111. doi: 10.1016/j.cities.2024.105111

[31] Chen M Cai Y Guo S Sun R Song Y Shen X. Evaluating implied urban nature vitality in San Francisco: an interdisciplinary approach combining census data, street view images, and social media analysis. Urban For Urban Green. (2024) 95:128289. doi: 10.1016/j.ufug.2024.128289

[32] Ma X Zhang Z Song Y. Geographically informed graph neural networks. Spat Stat. (2025) 69:100920. doi: 10.1016/j.spasta.2025.100920

[33] Li S Li W Chen L Jiang H Zhang J Gao DW. Real-time robust state estimation for large-scale low-observability power-transportation system based on meta physics-informed graph TimesNet. IEEE Trans Smart Grid. (2024) 15:5500–13. doi: 10.1109/TSG.2024.3408640, 32217482

[34] Wang L Liu X Zheng H Caneparo L. Quality improvement of urban public space from the perspective of the flâneur. Land. (2024) 13:808. doi: 10.3390/land13060808

[35] Xiao Y Li C Zhou Z Hou D Zhou X. Analysis and optimization of the spatial patterns of commercial service facilities based on multisource spatiotemporal data and graph neural networks: a case study of Beijing, China. IJGI. (2025) 14:23. doi: 10.3390/ijgi14010023

[36] Zhang Z Sun T Fisher T Wang H. The relationships between the campus built environment and walking activity. Sci Rep. (2024) 14:20330. doi: 10.1038/s41598-024-69881-8, 39223190 PMC11368923

[37] Chen L Zhao L Xiao Y Lu Y. Investigating the spatiotemporal pattern between the built environment and urban vibrancy using big data in Shenzhen, China. Comput Environ Urban Syst. (2022) 95:101827. doi: 10.1016/j.compenvurbsys.2022.101827

[38] Song Y Zhang W Deng Y Mo H Li Y. Decoding spatial vitality in historic districts: a grey relational analysis of multidimensional built environment factors in Shanghai’s Zhangyuan. Land. (2025) 14:1869. doi: 10.3390/land14091869

[39] Zhu J Lu H Zheng T Rong Y Wang C Zhang W . Vitality of urban parks and its influencing factors from the perspective of recreational service supply, demand, and spatial links. IJERPH. (2020) 17:1615. doi: 10.3390/ijerph17051615, 32131523 PMC7084482

[40] Kang W Oshan TM. Scale and correlation in multiscale geographically weighted regression (MGWR). J Geogr Syst. (2025) 27:399–424. doi: 10.1007/s10109-025-00468-1

[41] Sakai T Tsuchida J Yadohisa H. Bayesian geographically weighted regression using fused lasso prior. Spat Stat. (2025) 66:100884. doi: 10.1016/j.spasta.2025.100884

[42] Liu M Li L Li Q Bai Y Hu C. Pedestrian flow prediction in open public places using graph convolutional network. Int J Geo-Inf. (2021) 10:455. doi: 10.3390/ijgi10070455

[43] Nejadshamsi S Bentahar J Eicker U Wang C Jamshidi F. A geographic-semantic context-aware urban commuting flow prediction model using graph neural network. Expert Syst Appl. (2025) 261:125534. doi: 10.1016/j.eswa.2024.125534

[44] Li S Lai J Li H Tang W Xue Y Jiang H. CarbonGPT: meta causal graph-enhanced large language models for carbon emission forecasting in large-scale power distribution networks. IEEE Trans Smart Grid. (2026) 17:2797–809. doi: 10.1109/TSG.2026.3660950

[45] Sevtsuk A Kalvo R. Modeling pedestrian activity in cities with urban network analysis. Environ Plan B Urban Anal City Sci. (2025) 52:377–95. doi: 10.1177/23998083241261766

[46] Huang X Zeng L Liang H Li D Yang X Zhang B. Comprehensive walkability assessment of urban pedestrian environments using big data and deep learning techniques. Sci Rep. (2024) 14:26993. doi: 10.1038/s41598-024-78041-x, 39506013 PMC11541575

[47] Hou J Zhang E Long Y. Measuring pedestrian flows in public spaces: inferring walking for transport and recreation using Wi-Fi probes. Build Environ. (2023) 230:109999. doi: 10.1016/j.buildenv.2023.109999

[48] Huang B Mao G Qin Y Wei Y. Pedestrian flow estimation through passive WiFi sensing. IEEE Trans Mob Comput. (2021) 20:1529–42. doi: 10.1109/TMC.2019.2959610

[49] Jundee T Phithakkitnukoon S Ratti C. Inferring trips and origin-destination flows from Wi-Fi probe data: a case study of campus Wi-Fi network. IEEE Access. (2023) 11:63351–64. doi: 10.1109/ACCESS.2023.3288283

[50] Chen L Li Q Jia X Tang T. Field study on students’ walking behavior on stairways in university buildings. Saf Sci. (2025) 192:106997. doi: 10.1016/j.ssci.2025.106997

[51] Göçer Ö Göçer K Özcan B Bakovic M Kıraç MF. Pedestrian tracking in outdoor spaces of a suburban university campus for the investigation of occupancy patterns. Sustain Cities Soc. (2019) 45:131–42. doi: 10.1016/j.scs.2018.11.006

[52] Bonthu B Mohan S. Combining Wi-Fi fingerprinting and pedestrian dead reckoning to mitigate external factors for a sustainable indoor positioning system. Sustainability. (2023) 15:10943. doi: 10.3390/su151410943

[53] Zhang P Chen R Li Y Niu X Wang L Li M . A localization database establishment method based on crowdsourcing inertial sensor data and quality assessment criteria. IEEE Internet Things J. (2018) 5:4764–77. doi: 10.1109/JIOT.2018.2817599

[54] Liu S Zhu Z Gao Y Wang S. Assessing the dynamic vitality of public spaces in tourism-oriented traditional villages: a collaborative active perception method. Herit Sci. (2024) 12:346. doi: 10.1186/s40494-024-01467-z

[55] Xiang K Liu Y Huang Y Xiang S Lucchi E. Impact of built environment on behavioral vitality in transitional spaces: a modeling approach for university buildings in hot-humid climates. Energ Buildings. (2026) 366:117689. doi: 10.1016/j.enbuild.2026.117689

[56] Yang H Yao XA Whalen CC Kiwanuka N. Exploring human mobility: a time-informed approach to pattern mining and sequence similarity. Int J Geogr Inf Sci. (2025) 39:627–51. doi: 10.1080/13658816.2024.2427258, 40093814 PMC11906185

[57] Toprakli AY Satir MS. Urban mobility in Istanbul: unveiling gender and group dynamics in pedestrian walking speed. Int J BES. (2025) 12:41–57. doi: 10.11113/ijbes.v12.n2.1343

[58] Ewing R Handy S. Measuring the unmeasurable: urban design qualities related to walkability. J Urban Des. (2009) 14:65–84. doi: 10.1080/13574800802451155

[59] Gerike R Koszowski C Schröter B Buehler R Schepers P Weber J . Built environment determinants of pedestrian activities and their consideration in urban street design. Sustainability. (2021) 13:9362. doi: 10.3390/su13169362

[60] Peng Y Li W Nijhuis S Yu Y Wu Z. Seeing heritage through green and blue: assessing the visual influence of blue-green infrastructure (BGI) in historic urban areas (HUAs). Environ Impact Assess Rev. (2026) 118:108301. doi: 10.1016/j.eiar.2025.108301

[61] Duan J Wang H Liu L Zhang J. The attraction gradient of urban functions: how does functional mix at multiple scales predict urban vitality. Cities. (2025) 156:105516. doi: 10.1016/j.cities.2024.105516

[62] Jain J. Li J. Chiu M. Hassani A. Orlov N. Shi H. OneFormer: one transformer to rule universal image segmentation. In: Proceedings of the 2023 IEEE/CVF Conference on Computer Vision and Pattern Recognition (CVPR). IEEE. (2023) 2989–98. doi: 10.1109/CVPR52729.2023.00292

[63] Iamtrakul P Chayphong S Panuwatwanich K. A comprehensive exploration of urban spatial economic vitality through the application of the node-place model. J Urban Des. (2026) 31:179–93. doi: 10.1080/13574809.2025.2495913

[64] Lou G Chen Q Chen W. Strategic planning for sustainable urban park vitality: spatiotemporal typologies and land use implications in Hangzhou’s Gongshu District via multi-source big data. Land. (2025) 14:1338. doi: 10.3390/land14071338

[65] Li S Wu L Peng T Huang J Jiang H Xue Y . RSynLLM: a risk-aware routing mixture-of-experts large language model for multi-energy load forecasting in large-scale distribution networks. Appl Energy. (2026) 406:127227. doi: 10.1016/j.apenergy.2025.127227

[66] Li S Li H Li X Xu Y Lin Z Jiang H. Causal intervention is what large language models need for spatio-temporal forecasting. *IEEE Trans Cybern*. (2025) 55:3825–37. doi: 10.1109/TCYB.2025.3569333 40440153

[67] Li S Chen L Li W Chen G Kang J Niyato D . Meta-guided graph lightweight TimesNet for traffic prediction in Internet of Vehicles. IEEE Internet Things J. (2026) 13:22756–69. doi: 10.1109/JIOT.2026.3663375

[68] Li Q Han Z Wu X-M. Deeper insights into graph convolutional networks for semi-supervised learning. Proc AAAI Conf Artif Intell. (2018) 32:3538–45. doi: 10.1609/aaai.v32i1.11604

[69] Ma M Zhao H Wang J Lai Y. Urban multi-scale information graph and fusion model. Urban Inform. (2026) 5:7. doi: 10.1007/s44212-025-00097-5

[70] Nandan M Mitra S De D. GraphXAI: a survey of graph neural networks (GNNs) for explainable AI (XAI). Neural Comput Appl. (2025) 37:10949–11000. doi: 10.1007/s00521-025-11054-3

[71] Shan R Ning H Xu Q Su X Guo M Jia X. Physics-informed and explainable graph neural networks for generalizable urban building energy modeling. Appl Sci. (2025) 15:8854. doi: 10.3390/app15168854

[72] Liu X Li Z He B-J. Synergistic risks of summertime extreme heat and air pollution: evidence from a suburban area in Shanghai, China. Urban Clim. (2026) 68:103081. doi: 10.1016/j.uclim.2026.103081

